# Modeling Zn_3_O_3_/Ga_3_O_3_ composite for water splitting: a first-principle DFT study of electronic structure and interfacial reactivity

**DOI:** 10.1038/s41598-026-58040-w

**Published:** 2026-06-26

**Authors:** Hayat H. El-Agamy, Nada A. Khaled, Asmaa Ibrahim, Hanan Elhaes, Medhat A. Ibrahim

**Affiliations:** 1 Nuclear Materials Authority (NMA), P.O. Box 530, El-Maadi, Cairo, Egypt; 2 Therapeutic Chemistry Department, Pharmaceutical and Drug Industries Research Institute, National Research Centre, 33 El-Bohouth St., Dokki, Giza, 12622 Egypt; 3 Physics Department, Faculty of Women for Arts, Science and Education, Ain Shams University, Cairo, 11757 Egypt; 4 Spectroscopy Department, National Research Centre, 33 El-Bohouth St, Dokki, Giza, 12622 Egypt; 5 Molecular Modeling and Spectroscopy Laboratory, Centre of Excellence for Advanced Science, National Research Centre, 33 El-Bohouth St, Dokki, Giza, 12622 Egypt; 6 Center for Converging Sciences and Emerging Technologies (CoSET), Benha National University (BNU), El-Obour, 13518 Egypt

**Keywords:** Density functional theory (DFT), Zn₃O₃/Ga₃O₃ composite, Water splitting, HOMO-LUMO energy gap, Interfacial reactivity and molecular electrostatic potential (MESP), Chemistry, Materials science

## Abstract

This study employs Density Functional Theory (DFT) at the B3LYP/LANL2DZ level to investigate the structural and electronic properties of a Zn_3_O_3_/Ga_3_O_3_ composite and its interaction with water clusters. Using the Gaussian 09 suite, the research analyzes model molecules of Zn_3_O_3_, Ga_3_O_3_, and their composite to evaluate kinetic stability and chemical reactivity through HOMO-LUMO energy gaps and Total Dipole Moments (TDM). The results demonstrate that the Zn_3_O_3_/Ga_3_O_3_ composite is a tunable nanostructure whose electronic properties are highly sensitive to its interface and the presence of external moisture. The study used Molecular Electrostatic Potential (MESP) and Non-Covalent Interaction (NCI) methods to show that water adsorption becomes more stable through the combined effect of strong metal-oxygen coordination and hydrogen bonding and weak van der Waals forces. The Density of States (DOS) analysis demonstrated that the electronic structure and interfacial stability both changed because of the observed modulation. These findings provide critical insights into the initial steps of photocatalytic water splitting and suggest the composite as a promising candidate for water capture and catalytic applications. These results indicate that interfacial electronic coupling between Zn and Ga oxide domains enhances water affinity and electronic responsiveness, representing a key step in photocatalytic water splitting mechanisms. The findings provide atomistic insight into how heterostructured oxide composites can be rationally engineered to improve water capture, charge separation, and catalytic efficiency, highlighting the Zn₃O₃/Ga₃O₃ composite as a promising model for next-generation photocatalytic water splitting by optimizing surface adsorption and interfacial electronic coupling.

## Introduction

 Photocatalytic water splitting stands out as a sustainable, clean, and promising method for energy conversion and hydrogen production, with the potential to address critical energy and environmental challenges facing modern society. The process typically involves a material that acts as a catalyst. When this material is exposed to sunlight, it undergoes photon absorption, charge separation followed by surface redox reactions (reduction/oxidation)^1–5^.

Zinc oxide (ZnO) is a multifunctional material with a wide range of applications. ZnO’s diverse crystal structures and morphologies, combined with its unique electronic, optical, and surface properties, underpin its extensive range of applications across electronics, optoelectronics, energy, environmental, and biomedical fields^6–9^.

Gallium oxide (GaO), particularly in its β phase, is a versatile material with a unique combination of wide bandgap, high stability, and tunable properties, enabling its use in a wide array of electronic, optoelectronic, sensing, and catalytic applications^10–12^.

Composites of ZnO and Ga₂O₃ can be formed using various deposition and synthesis methods, resulting in materials with tunable optical and electrical properties. These composites are applied in optoelectronics, photocatalysis, sensing, and display technologies, with their performance and application scope determined by their structural and compositional characteristics^13–15^.

Zn₃O₃ and Ga₃O₃ are referenced in the literature primarily as components or motifs within zinc oxide (ZnO) and gallium oxide (Ga₂O₃) materials, which are widely recognized for their roles in catalysis and electronics. These oxides, often forming spinel or other crystalline structures, serve as primary clusters or building blocks in advanced material systems for various applications. Zn₃O₃ and Ga₃O₃ clusters, as realized in ZnO and Ga₂O₃ materials, are established as primary building blocks in catalysis and electronics, with their structural, electronic, and defect properties being central to their performance in these fields^16–18^.

The integration of ZnO and Ga_2_O_3_ into a composite overcomes the limitations of using either oxide alone. By leveraging a tunable nanostructure and increased interfacial activity, this combination provides the high surface area and porosity necessary for high-performance moisture sensing. Ultimately, this material synergy allows for a more responsive and customizable sensor than its individual components^19–21^.Ga³⁺ doping in ZnO-based catalysts leads to strong interactions between Zn and Ga species, with partial electron transfer from Zn to Ga. Ga exists in several forms, including framework tetrahedral Ga³⁺, extra-framework Ga³⁺ bonded to oxygen, and Ga₂O₃ clusters. High Ga content results in the formation of Ga₂O₃ clusters, which are directly evidenced by XPS analysis^22^.

Gallium oxide serves as a highly tunable platform for CO₂ conversion. Its performance is governed by two main factors: the crystalline phase (which determines the reaction pathway) and the synergy with metal additives like Pd and Zn. By selecting the optimal polymorph and promoting the formation of active alloys like PdZn, researchers can precisely direct the catalytic process toward desired products like methanol or light olefins^23^.

Density Functional Theory (DFT) serves as a cornerstone of modern computational physics and chemistry, providing a sophisticated method for analysing many-electron systems. It could provide several parameters for many systems whereas experimental data is limited and/or unavailable^24–27^.

DFT is widely used to investigate the electronic, structural, and optical properties of complex materials, including ZnO and GaO, as well as their doped and composite forms. DFT enables the calculation of charge distribution, and defect states, which are essential for understanding and optimizing material performance in various applications such as sensors, photocatalysts, and optoelectronic devices^28–31^.

Recent studies have demonstrated that heterostructured oxide composites are an effective strategy for enhancing photocatalytic performance through interfacial band alignment and improved charge separation^32^. ZnO-based heterojunctions combined with Ga₂O₃ or other Ga-containing oxides have exhibited higher photocurrent response, increased water adsorption, and greater catalytic stability due to synergistic electronic interactions at the interface^33^. Advances in nanoscale engineering have further highlighted the critical role of interfacial electronic redistribution, defect modulation, and surface polarization in controlling adsorption and reaction pathways during photocatalytic water splitting^34^. Nevertheless, despite these experimental advances, the atomistic mechanisms governing early-stage water adsorption and electronic coupling at Zn–Ga oxide interfaces remain incompletely understood, particularly at the molecular cluster level where catalytic activation is initiated.

Both ZnO and GaO systems show that doping (with metals or non-metals) can be used to tailor electronic and optical properties, such as band gap reduction, increased absorption, and modified carrier mobility. The specific effects depend on the dopant’s electronegativity, ionic size, and bonding preferences^35^.

The observed photocurrent densities for GaON, ZnO, and GaON/ZnO were approximately 0.2, 0.4, and 1.2 mA/cm², respectively. This indicates a significant enhancement (6-fold over GaON and 3-fold over ZnO) in the photoelectrochemical water splitting performance for the GaON/ZnO nanoarchitecture^36^. DFT studies on ZnO/GaON heterostructures reveal that the combination of ZnO clusters with GaON surfaces leads to strong water adsorption and enhanced dissociative adsorption energy, which are crucial for efficient water splitting.

The aim of this research is to utilize first-principles DFT at the B3LYP/LANL2DZ level to investigate the structural, electronic, and interfacial properties of a Zn_3_O_3_/Ga_3_O_3_ composite cluster, specifically focusing on its potential for early-stage water splitting activation. By analyzing the model molecules through the Gaussian 09 suite, the study seeks to evaluate the kinetic stability and chemical reactivity of the composite compared to its individual components, Zn_3_O_3_ and Ga_3_O_3_, using HOMO-LUMO energy gaps and Total Dipole Moments (TDM). Furthermore, the work aims to characterize the interfacial reactivity and the mechanisms of water adsorption by employing Molecular Electrostatic Potential (MESP). Non-Covalent Interaction (NCI) analyses to understand how metal-oxygen coordination and hydrogen bonding stabilize the interaction with moisture. Also, the Density of States DOS. Ultimately, this work intends to provide fundamental theoretical insights into the initial steps of water capture and catalytic activation, positioning the Zn_3_O_3_/Ga_3_O_3_ nanostructure as a viable candidate as surface activation for sustainable energy conversion technologies.

## Computational details

**Fig. 1:**
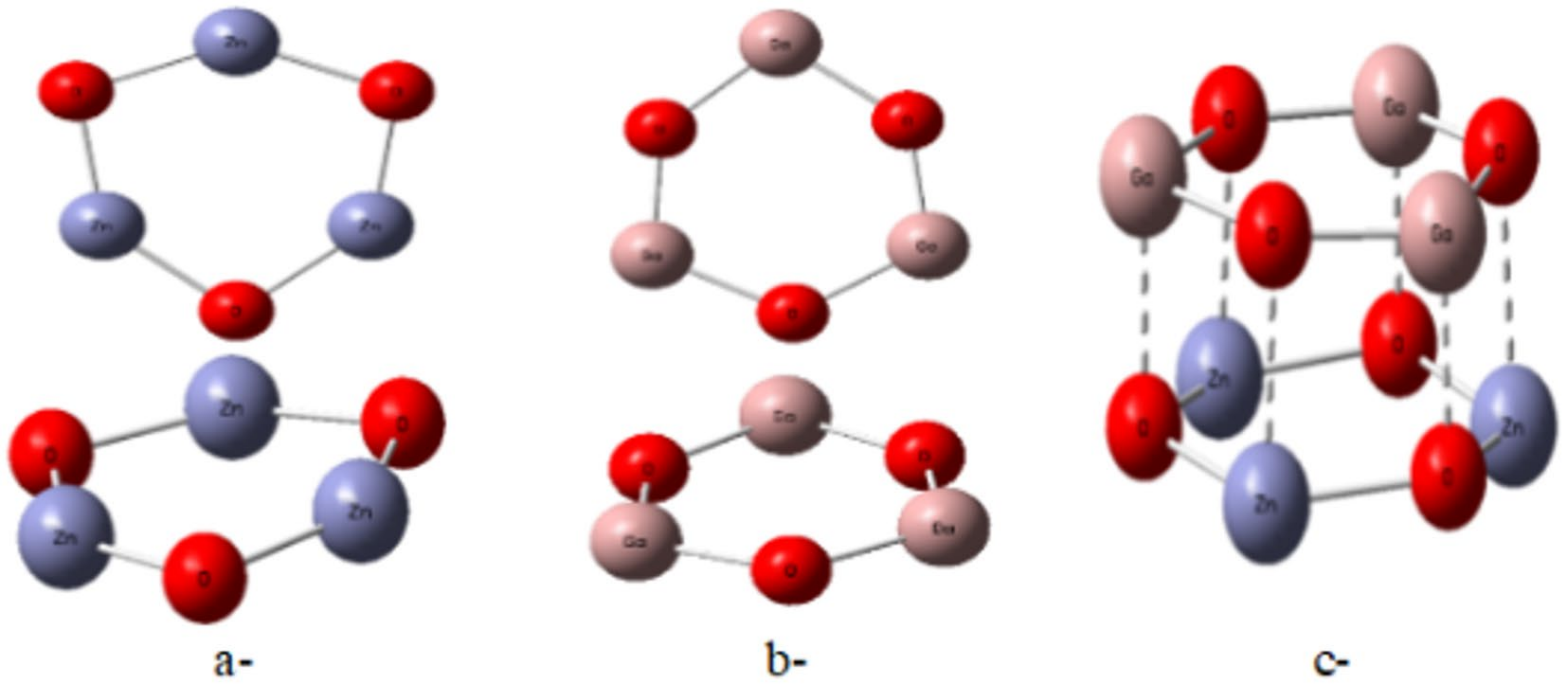
The studied model molecules (**a**) Zn_3_O_3_ (as elevation and plan), (**b**) Ga_3_O_3_ (as elevation and plan) and (**c)** Zn_3_O_3_/Ga_3_O_3_ composite.

A model molecule of Zinc oxide Zn_3_O_3_ is indicated in Figure 1a, then Gallium oxide Ga_3_O_3_ is shown as in Figure 1b. A model molecule for Zn_3_O_3_/Ga_3_O_3_ composite is shown in Figure 1c.

Molecular modeling calculations were performed using the Gaussian 09 suite of programs^37^. Initial molecular structures were constructed using the GaussView 6.0^38^ graphical interface. Geometry optimizations and vibrational frequency analyses were carried out using Density Functional Theory (DFT) with the B3LYP hybrid functional^39–41^. This functional, which incorporates Becke’s three-parameter exchange and the Lee-Yang-Parr correlation functional, was employed in conjunction with the LANL2DZ (Los Alamos National Laboratory 2-double-zeta) basis set for all atoms. The LANL2DZ basis set utilizes Effective Core Potentials (ECP) to represent chemically inert core electrons. This approach is particularly advantageous for zinc (Zn) as it reduces computational overhead while accounting for scalar relativistic effects. All stationary points were confirmed as local minima on the potential energy surface by the absence of imaginary frequencies (number of imaginary frequencies = 0). Optimizations were performed using the Berny algorithm and continued until all standard convergence criteria were satisfied, specifically: maximum force, root-mean-square (RMS) force, maximum displacement, and RMS displacement. To facilitate direct comparison with experimental data, Raman activities were calculated analytically at the same level of theory. Molecular connectivity was explicitly maintained throughout the optimization process using the geom=connectivity keyword.

Within the same theoretical framework, the Total Dipole Moment (TDM) and frontier molecular orbital energies specifically the Highest Occupied Molecular Orbital (HOMO) and Lowest Unoccupied Molecular Orbital (LUMO) were evaluated. The HOMO-LUMO energy gap (ΔE) was calculated as follows:

$${\mathbf{\Delta E}} = {\mathrm{E}}_{{{\mathrm{LUMO}}}} - {\mathrm{E}}_{{{\mathrm{HOMO}}}}$$

The HOMO-LUMO gap and TDM measurements function as indicators which determine the electronic reactivity and kinetic stability of the Ga_3_O_3_/Zn_3_O_3_ systems. Molecular Electrostatic Potential (MESP) and Non-Covalent Interaction (NCI) analyses provided us with detailed localized bonding environment descriptions which we used to interpret how water molecules interact with other molecules and which types of interactions occur. NBO analyses were also studied. The NBO evaluation was estimated by NBO 3.1^42^.

To provide a deeper insight into the electronic structure, the Frontier molecular orbitals are commonly utilized in conjunction with Koopman’s theorem to determine values for ionization energy and electron affinity, expressed as I = – E (HOMO) and A = – E (LUMO). Nevertheless, this approach overlooks the effects of orbital relaxation and electron correlation corrections. To overcome this deficiency, calculations have been performed for the energies of neutral (N), cationic (N – 1), and anionic (*N* + 1) species for each compound. Consequently, the ionization energy and electron affinity were determined using I = E (N – 1) – E (N), and A = E (N) – E (N + 1), thus avoiding the previously mentioned limitations. The additional chemical descriptors are evaluated through the following equations: µ = – χ ≅ – I + A /2, η ≅ I – A, ω = χ2 / 2η, and ε = 1/ ω, which correspond to chemical potential, chemical hardness, electrophilicity index, and nucleophilicity respectively^43,44^.These global reactivity indices were calculated using Multiwfn software.

Finally, the electronic wavefunction was exported in WFN format to enable subsequent electron-density and bonding analyses using Multiwfn, including Quantum Theory of Atoms in Molecules (QTAIM)^45^ and Non-Covalent Interaction (NCI) analyses^46^. The molecular electrostatic potential (MESP)^47^ was also calculated from the WFN file using the Multiwfn program, and all topological and electrostatic results were visualized using VMD^48,49^.

The nature of the interactions between water molecules and the nanocomposite was analyzed within the framework of the Quantum Theory of Atoms in Molecules (QTAIM), enabling evaluation of the electronic characteristics and interaction types governing the hydrated complexes. QTAIM analysis was performed using the topology of the electron density to identify nuclear critical points (NCPs) and bond critical points (BCPs). Nuclear critical point properties primarily reflect core-electron localization at atomic nuclei and were therefore used only to describe electronic distribution and structural symmetry within the clusters. In contrast, chemical bonding and intermolecular interaction characterization were derived exclusively from bond critical point descriptors. Bond classification was based on combined analysis of electron density ρ(r), Laplacian ∇²ρ(r), and total energy density H(r), allowing differentiation between shared-shell and closed-shell interactions according to established QTAIM criteria.

To further elucidate the interfacial chemistry, Non-Covalent Interaction (NCI) and Reduced Density Gradient (RDG) analyses were employed. These methods allowed for the visualization and mapping of weak interaction regions, offering a comprehensive description of hydrogen bonding, π-stacking, and van der Waals forces at the interface with both the composite and surrounding water molecules.

## Results and discussions

**Fig. 2:**
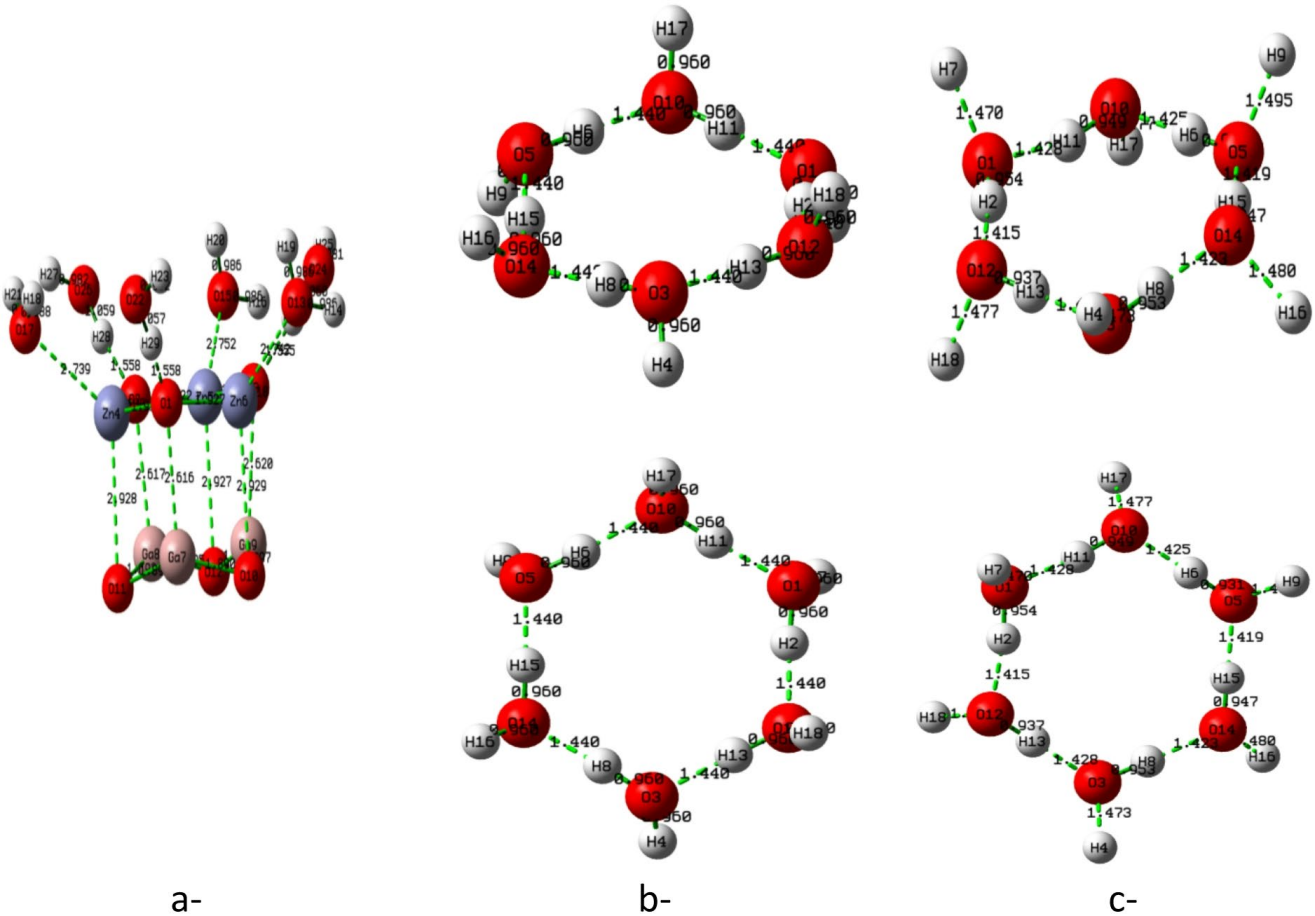
The model structure of (**a**) Zn_3_O_3_/Ga_3_O_3_ composite interacting with 6 water molecules, (**b**) cluster of 6 water molecules (as elevation and plan) and (**c**) cluster of 6 water molecules whereas splitting started (as elevation and plan).

Figure 2a presents the Zn₃O₃/Ga₃O₃ composite interacting with a water hexamer cluster positioned near the surface. Figure 2b shows a plan view of a hexagonal or ring arrangement, a stable configuration for a 6-water molecule cluster. The elevation view illustrates the vertical orientation and the buckling of the water molecules, highlighting the hydrogen bonding network. Finally, Figure2c depicts the dissociation of water, representing the initial step in processes such as photocatalytic water splitting or surface hydroxylation.

As indicated in Figure 2, the composite model displays how the inorganic framework interacts with water molecules which exist in the system. The water molecules establish connections with the Zn atoms through bonds which display distances between 1.928 Å and 2.739 Å. The cluster maintains a consistent distance between Zn–O and Ga–O which measures approximately 2.617 Å. This behavior shows that water adsorption creates local polarization in the material without causing immediate structural failure of the composite. The internal O–H bond lengths maintain a constant measurement of 0.960 Å which represents the typical length for non-dissociated water. The inter-molecular hydrogen bonds (O–H) are measured at 1.440 Å. A hydrogen-bonding network which exhibits very strong attraction forms in clusters that exist either in vacuum spaces or in areas with limited movement because the distance between particles measures 1.440 Å which is shorter than the normal distance of 1.97 Å found in liquid water. Model c shows the moment when water molecules start to break apart through the process of dissociation. The bond lengths present a distinct shift. Some O–H covalent bonds have stretched from 0.960 Å to approximately 0.931–0.954 Å. The distances between model c elements exhibit high variability because they extend from 1.415 Å to 1.495 Å. The oxygen-proton distance measurements of 1.423 Å and 1.477 Å indicate that the system undergoes a proton-relay mechanism. The Zn_3_O_3_/Ga_3_O_3_ surface creates a “computational signature” which acts as a catalytic effect by reducing the energy required for O–H bond breakage.

Table 1 summarizes the calculated total dipole moment (TDM) and HOMO–LUMO energy gap (ΔE) of the investigated systems. Significant variations in both parameters were observed upon heterostructure formation and water adsorption, highlighting the strong interfacial electronic coupling within the Zn₃O₃/Ga₃O₃ nanocomposite.

The isolated Zn₃O₃ cluster exhibits a very small dipole moment (0.0184 D), indicating a nearly symmetric charge distribution, together with a wide energy gap (3.76 eV), characteristic of relatively low chemical reactivity and semiconducting behavior. In contrast, Ga₃O₃ shows a higher dipole moment (0.2378 D) and a significantly narrower energy gap (1.36 eV), suggesting enhanced charge polarization and higher electronic softness.

**Fig. 3:**
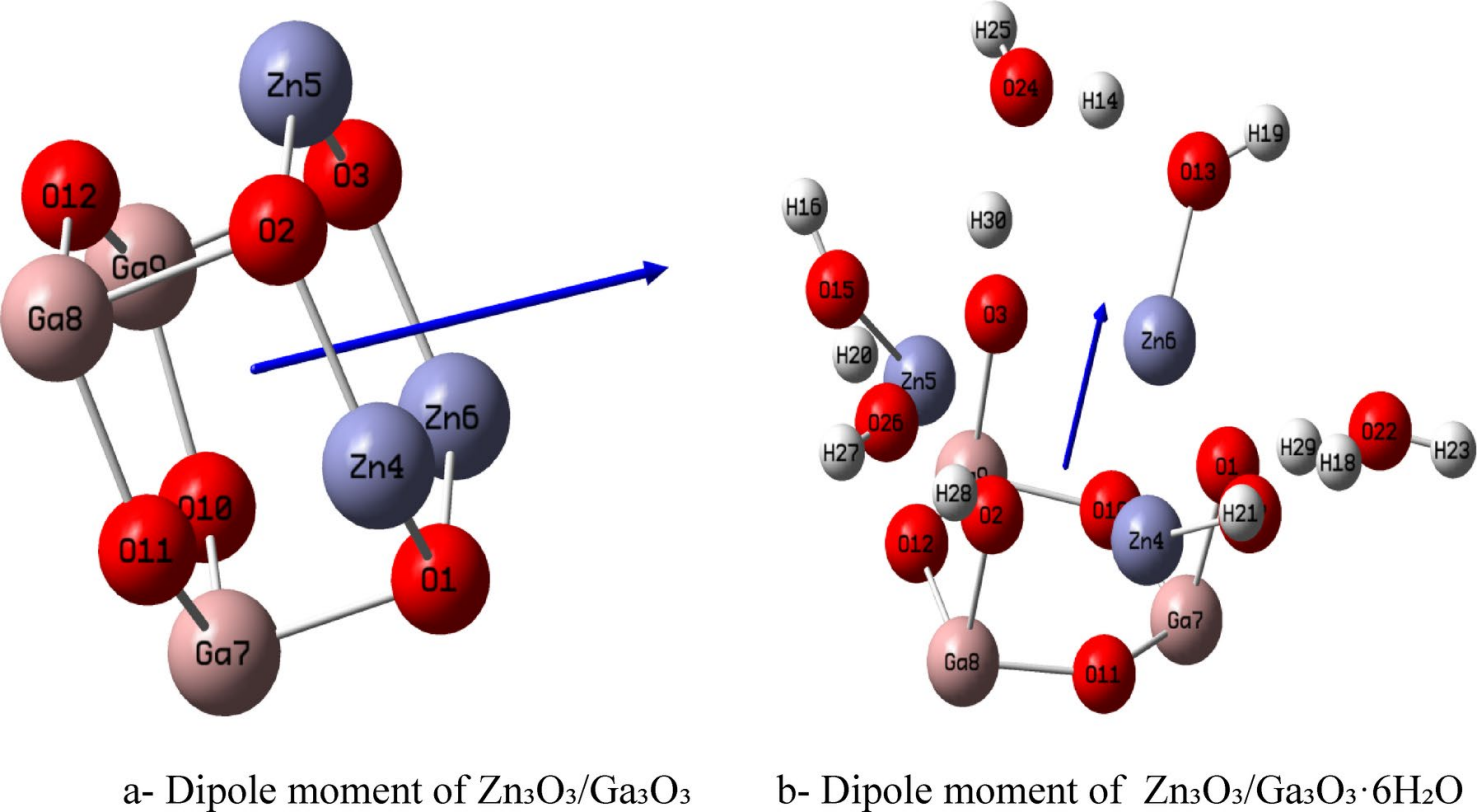
Dipole moment vectors of (**a**) Zn₃O₃/Ga₃O₃ heterostructure and (**b**) hydrated Zn₃O₃/Ga₃O₃·6 H₂O complex. The blue arrows represent the magnitude and direction of the total dipole moment.

Upon formation of the Zn₃O₃/Ga₃O₃ interface, the dipole moment increases sharply to 3.55 D, confirming substantial charge redistribution at the heterointerface, as further supported by the pronounced dipole vector shown in Figure 3a. Simultaneously, the energy gap decreases to 1.33 eV, indicating improved electronic conductivity and enhanced reactivity compared to pristine Zn₃O₃. Such band-gap narrowing upon heterostructure formation is consistent with previously reported oxide-based nanocomposites, where interfacial orbital hybridization promotes charge transfer and electronic delocalization.

Upon adsorption of six water molecules, the energy gap increases to 2.34 eV, accompanied by a reduction in dipole moment to 2.16 D and a clear attenuation and reorientation of the dipole vector (Figure 3b). This suggests that hydration partially suppresses interfacial charge transfer and reduces electronic softness. Consequently, the dipole vector becomes less pronounced and deviates from its original direction in the dry heterostructure. Therefore, moisture exposure may significantly alter the electronic performance of the Zn₃O₃/Ga₃O₃ system. For electronic or catalytic applications requiring narrow band gaps and high reactivity, environmental humidity control may be essential to preserve optimal performance.

Overall, these results demonstrate that the Zn₃O₃/Ga₃O₃ nanostructure exhibits tunable electronic properties strongly governed by interfacial effects and external molecular adsorption.

**Table 1:** Calculated total dipole moment (TDM) and HOMO–LUMO energy gap (ΔE) of isolated clusters, Zn₃O₃/Ga₃O₃ heterostructure, and hydrated complexes, illustrating interfacial and moisture-induced modulation of electronic properties.

Structures	TDM (Debye)	ΔE (eV)
Zn_3_O_3_	0.0184	3.7587
Ga_3_O_3_	0.2378	1.3606
Zn_3_O_3_/Ga_3_O	3.5529	1.3312
Zn_3_O_3_/Ga_3_O_3_.6H_2_O	2.1610	2.3392
6H_2_O	0.0045	9.8433
6 H……OH	0.0039	9.8430

**Fig. 4:**
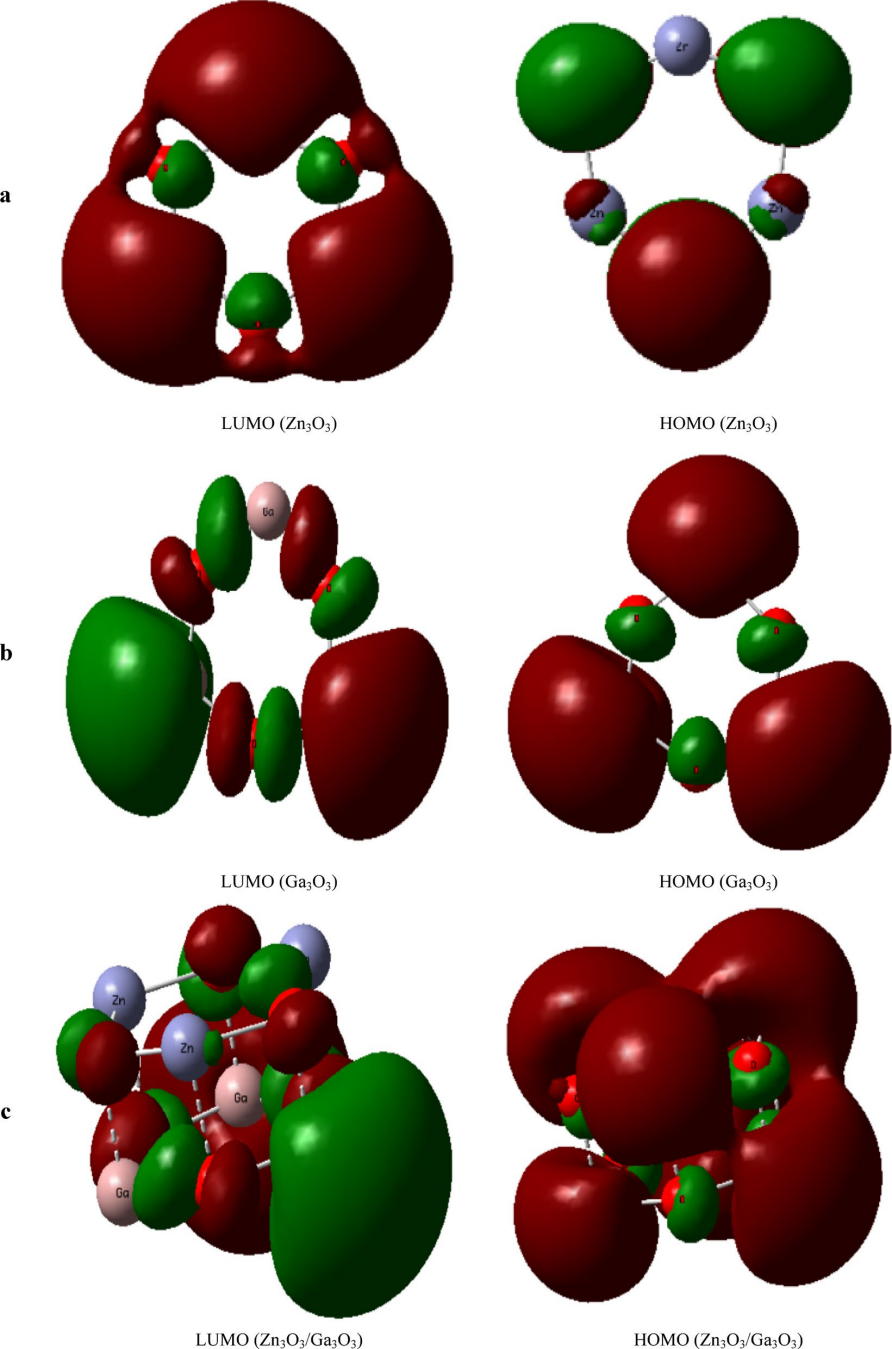

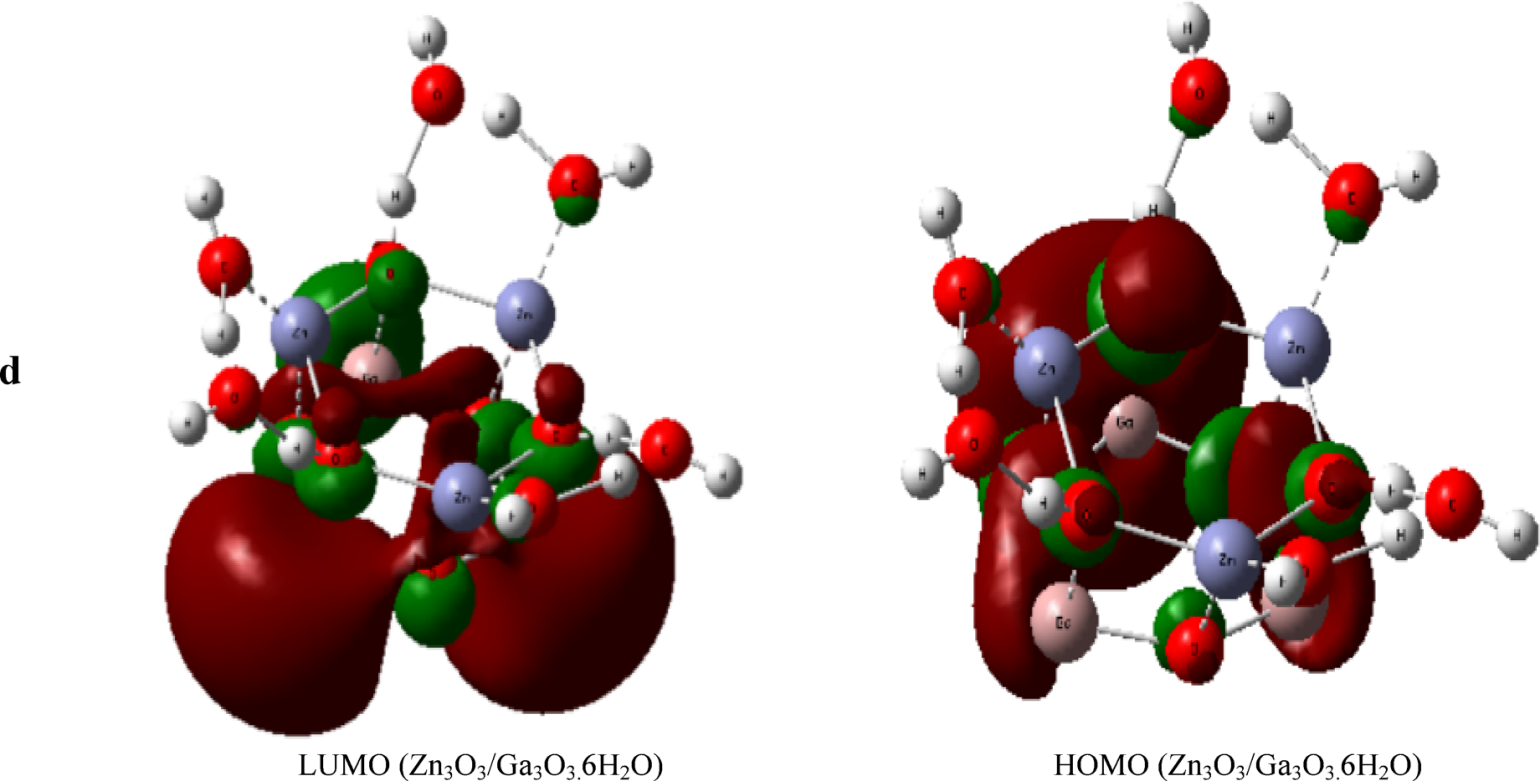
HOMO/LUMO for (**a**) Zn_3_O_3_, (**b**) Ga_3_O_3_ (**c**) Zn_3_O_3_/Ga_3_O_3_ composite and (**d**) Zn_3_O_3_/Ga_3_O_3_ composite interacting with 6 water molecules.

Figure 3 provides a comparative visualization of the Highest Occupied Molecular Orbitals (HOMO) and Lowest Unoccupied Molecular Orbitals (LUMO) across four distinct molecular configurations. These orbital distributions are essential for evaluating electronic properties, chemical reactivity, and light-absorption potential in materials science. The Ffigure breaks down the electronic evolution from individual components to a complex hydrated system:

As shown in Figure 4a and b, the isolated zinc oxide Zn_3_O_3 _and gallium oxide Ga_3_O_3_ clusters exhibit orbitals that are relatively symmetric and localized around their respective ring structures. As shown in Figure 4c, as far as these clusters merge into Zn_3_O_3_/Ga_3_O_3_ composite, the electronic states shift upon the formation of the heterojunction. Notably, the LUMO exhibits significant localization on one side of the structure, suggesting an inherent spatial separation of charges. Hydrated state which is indicated in Figure 4d, shows that, the interaction with six water molecules 6H_2_O causes a pronounced shift in orbital density. In this state, the LUMO becomes concentrated at the base of the cluster, while the HOMO distributes across the interface interacting with the water molecules. The observed spatial separation of the HOMO and LUMO in the composite and hydrated states is a key indicator of efficient electron-hole pair separation. This characteristic is critical for maximizing performance in photocatalytic applications, such as solar energy conversion or water splitting.

The global reactivity indices reveal distinct electronic characteristics for each composite system that are intrinsically related to their water adsorption capabilities (Table 2). The pristine Zn₃O₃ cluster exhibits the highest chemical hardness (η = 7.52 eV) and electrophilicity index (ω = 1.78 eV), coupled with the lowest softness (S = 0.13 eV⁻¹) and nucleophilicity index (ε = 0.56 eV), indicating a relatively rigid electronic structure with strong electron-accepting character that limits its polarizability and interaction with water molecules. In contrast, the Ga₃O₃ cluster demonstrates intermediate reactivity parameters (η = 5.87 eV, ω = 1.66 eV, S = 0.17 eV⁻¹, ε = 0.60 eV), suggesting a more balanced electronic distribution. The designed mixed oxide composite Zn₃O₃/Ga₃O₃ displays a remarkable transformation in reactivity descriptors, with the lowest chemical hardness (η = 4.86 eV), highest softness (S = 0.21 eV⁻¹), reduced ionization energy (I = 6.10 eV), and significantly enhanced nucleophilicity (ε = 0.72 eV). These modifications indicate that the composite possesses a highly polarizable and reactive electronic framework that facilitates favorable electrostatic interactions with polar water molecules through improved charge transfer dynamics. Upon hydration, the Zn₃O₃/Ga₃O₃·6 H₂O system exhibits a moderate increase in hardness (η = 6.10 eV) and a further enhancement in nucleophilic character (ε = 0.82 eV), alongside the lowest electrophilicity index (ω = 1.22 eV) and electron affinity (A = 0.81 eV) among all systems studied. This electronic reorganization upon water adsorption confirms that the composite design successfully creates Lewis acid-base interaction sites where the metal centers act as electron acceptors and the oxygen sites provide nucleophilic centers for hydrogen bonding with water molecules. The systematic decrease in chemical hardness and increase in softness from pristine clusters to the mixed oxide demonstrates that composite formation introduces electronic defects and heterojunctions that enhance surface reactivity, while the balanced electrophilicity-nucleophilicity profile of the hydrated system indicates stable water coordination without excessive charge transfer that could compromise structural integrity. These findings establish that the rational design of Zn₃O₃/Ga₃O₃ composites optimizes the electronic properties necessary for efficient water adsorption through synergistic effects that simultaneously enhance polarizability, moderate electron affinity, and create favorable binding sites for water molecules .

**Table 2:** Computed global reactivity parameters where E(N) represents the energy of the neutral species, E(N + 1) denotes the energy of the anionic species, E(N − 1) indicates the energy of the cationic species, I signifies ionization energy, A represents electron affinity, χ corresponds to electronegativity, µ denotes chemical potential, η indicates hardness, S represents softness, ω signifies the electrophilicity index, ε denotes the nucleophilicity index, and HA represents Hartree units.

	Zn_3_O_3_	Ga_3_O_3_	Zn_3_O_3_/Ga_3_O	Zn_3_O_3_/Ga_3_O_3_.6H_2_O
E(*N*)/HA	− 422.49	− 231.99	− 654.68	− 1113.50
E(*N* + 1)/HA	− 422.55	− 232.05	− 654.72	− 1113.53
E(N − 1)/HA	− 422.17	− 231.72	− 654.45	− 1113.25
I/eV	8.93	7.34	6.10	6.92
A/eV	1.42	1.48	1.25	0.81
χ/eV	5.18	4.41	3.68	3.86
µ/eV	− 5.18	− 4.41	− 3.68	− 3.86
η/eV	7.52	5.87	4.86	6.10
S/eV^−¹^	0.13	0.17	0.21	0.16
ω/eV	1.78	1.66	1.39	1.22
ε/eV	0.56	0.60	0.72	0.82

To assess the thermodynamic stability and physical feasibility of the proposed Zn₃O₃/Ga₃O₃ composite system, the formation energy was calculated following the approach outlined in the literature^50^. The formation energy is defined as:

$$\:{\Delta\:}{E}_{f}=E({\mathrm{Zn}}_{3}{\mathrm{O}}_{3}/{\mathrm{Ga}}_{3}{\mathrm{O}}_{3})-E\left({\mathrm{Zn}}_{3}{\mathrm{O}}_{3}\right)-E\left({\mathrm{Ga}}_{3}{\mathrm{O}}_{3}\right)$$

where $\:E({\mathrm{Zn}}_{3}{\mathrm{O}}_{3}/{\mathrm{Ga}}_{3}{\mathrm{O}}_{3})$, $\:E\left({\mathrm{Zn}}_{3}{\mathrm{O}}_{3}\right)$, and $\:E\left({\mathrm{Ga}}_{3}{\mathrm{O}}_{3}\right)$represent the total ground-state energies of the Zn₃O₃/Ga₃O₃ heterostructure, the isolated Zn₃O₃ cluster, and the isolated Ga₃O₃ cluster, respectively. The computed total energies are − 654.68 Ha for the composite, − 422.49 Ha for Zn₃O₃, and − 231.99 Ha for Ga₃O₃. The resulting formation energy is $\:{\Delta\:}{E}_{f}=-0.200$Ha (− 5.44 eV), a substantially negative value that unambiguously confirms the thermodynamic favorability of heterostructure formation. The negative sign of $\:{\Delta\:}{E}_{f}$indicates that the Zn₃O₃/Ga₃O₃ composite is energetically more stable than its isolated constituent clusters and therefore will not spontaneously decompose under equilibrium conditions. These findings are consistent with previously reported stability criteria for two-dimensional composite systems^50^ and provide strong evidence that the proposed Zn₃O₃/Ga₃O₃ heterostructure represents a physically realistic and chemically stable configuration suitable for further investigation of its electronic and optical properties.

The interaction strength between the Zn₃O₃/Ga₃O₃ composite and water molecules was quantitatively evaluated through binding energy calculations. The total binding energy was determined using the relation:

$$\begin{aligned} E_{{{\mathrm{bind(total)}}}} & = E_{{{\text{composite + 6H}}_{2} O}} - \left( {E_{{{\mathrm{composite}}}} + E_{{6H_{2} O}} } \right) \\ & = - 1113.332197 - \left( { - 654.655940 + ( - 458.487326)} \right) \\ & = - 0.188931\:{\mathrm{Ha}}( \approx - {\mathrm{5}}.{\mathrm{14}}\:{\mathrm{eV}}). \\ \end{aligned}$$

The negative binding energy confirms the thermodynamic favorability and spontaneous nature of water adsorption on the composite surface. Furthermore, the average binding energy per water molecule, calculated as:

$$E_{{{\mathrm{bind(avg)}}}} = \frac{{E_{{{\mathrm{bind(total)}}}} }}{6} = \frac{{ - 0.188931}}{6} = - 0.0315{\mathrm{Ha}}( \approx - {\mathrm{0}}.{\mathrm{86}}\:{\mathrm{eV}}),$$

indicates moderate and uniform interactions between the oxide framework and the adsorbed water cluster.

### Molecular electrostatic potential (MESP)

**Fig. 5:**
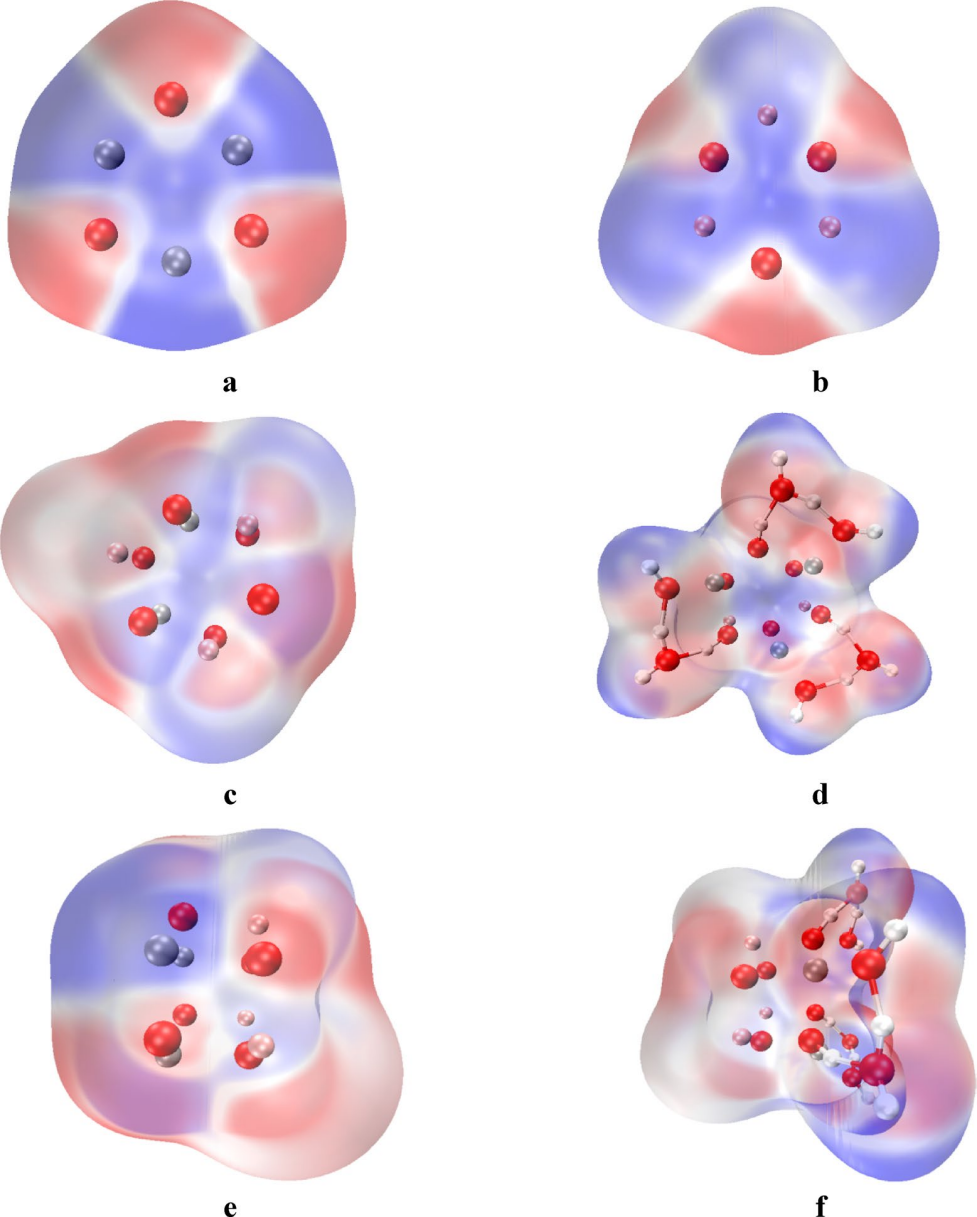
Mapping MESP for (**a**) Zn_3_O_3_, (**b**) Ga_3_O_3_ (**c**) Zn_3_O_3_/Ga_3_O_3_ composite (front orientation), (**d**) Zn_3_O_3_/Ga_3_O_3_ composite interacting with 6 water molecules (front orientation), (**e**) Zn_3_O_3_/Ga_3_O_3_ composite (side orination) and (**f**) Zn_3_O_3_/Ga_3_O_3_.6H_2_O (side ordination).

The molecular electrostatic potential (MESP) surfaces provide critical insights into the charge distribution and reactive sites of each composite system, which are fundamental to understanding their water adsorption mechanisms. For the pristine Zn₃O₃ cluster (Figure 5a), the MESP exhibits a highly symmetric distribution with distinct electronegative regions (red) localized on oxygen atoms and electropositive regions (blue) concentrated on zinc centers, creating well-defined alternating positive and negative potential zones that reflect the ionic character of the Zn–O bonds. The Ga₃O₃ cluster (Figure 5b) displays a similar but less pronounced electrostatic contrast, with moderately negative potentials on oxygen sites and positive potentials on gallium atoms, indicating a relatively more covalent bonding character compared to Zn₃O₃. The mixed oxide composite Zn₃O₃/Ga₃O₃ (Figure 5c and e) reveals a remarkable redistribution of electrostatic potential, characterized by an asymmetric surface topology with intensified negative potential regions and the emergence of intermediate potential zones at the Zn–O–Ga interface. This heterogeneous potential landscape creates multiple favorable interaction sites with varying electrostatic strengths, which is essential for accommodating water molecules through both strong Lewis acid-base interactions at metal centers and hydrogen bonding at oxygen sites. Upon hydration, the Zn₃O₃/Ga₃O₃·6 H₂O system (Figure 5d and f) exhibits a substantially modified MESP with expanded electronegative regions surrounding the adsorbed water molecules and a more diffuse positive potential distribution across the composite framework. The presence of water molecules significantly modulates the surface electrostatics by forming hydrogen-bonding networks that bridge the oxide framework, as evidenced by the continuous negative potential enveloping the hydration layer and the reduced potential gradients compared to the bare composite. From a design perspective, the MESP analysis demonstrates that the strategic combination of Zn₃O₃ and Ga₃O₃ generates complementary electrostatic environments where zinc sites provide strong Lewis acidic centers for water coordination through electron-deficient regions, while oxygen atoms in the composite offer nucleophilic sites with enhanced negative potential for hydrogen bonding with water protons. The asymmetric potential distribution in the mixed oxide composite, absent in the homogeneous pristine clusters, indicates that interfacial effects between different metal oxides create electrostatically favorable pockets and channels that can simultaneously accommodate multiple water molecules through cooperative binding mechanisms. Furthermore, the modulation of MESP upon hydration confirms that the composite design successfully balances strong initial water adsorption (evidenced by significant potential redistribution) with structural stability (maintaining distinct potential regions even after saturation with six water molecules), which is crucial for reversible water uptake and release in practical desiccant applications.

### Non-covalent interaction (NCI) analysis

**Fig. 6:**
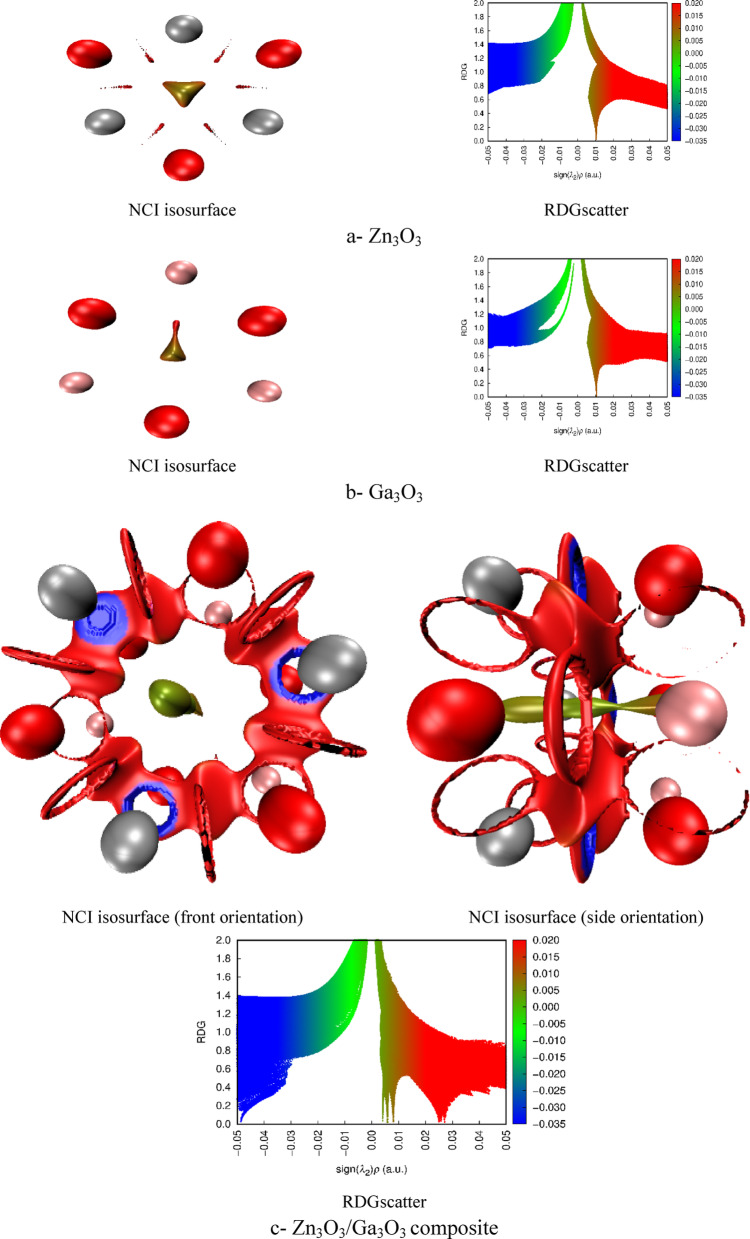

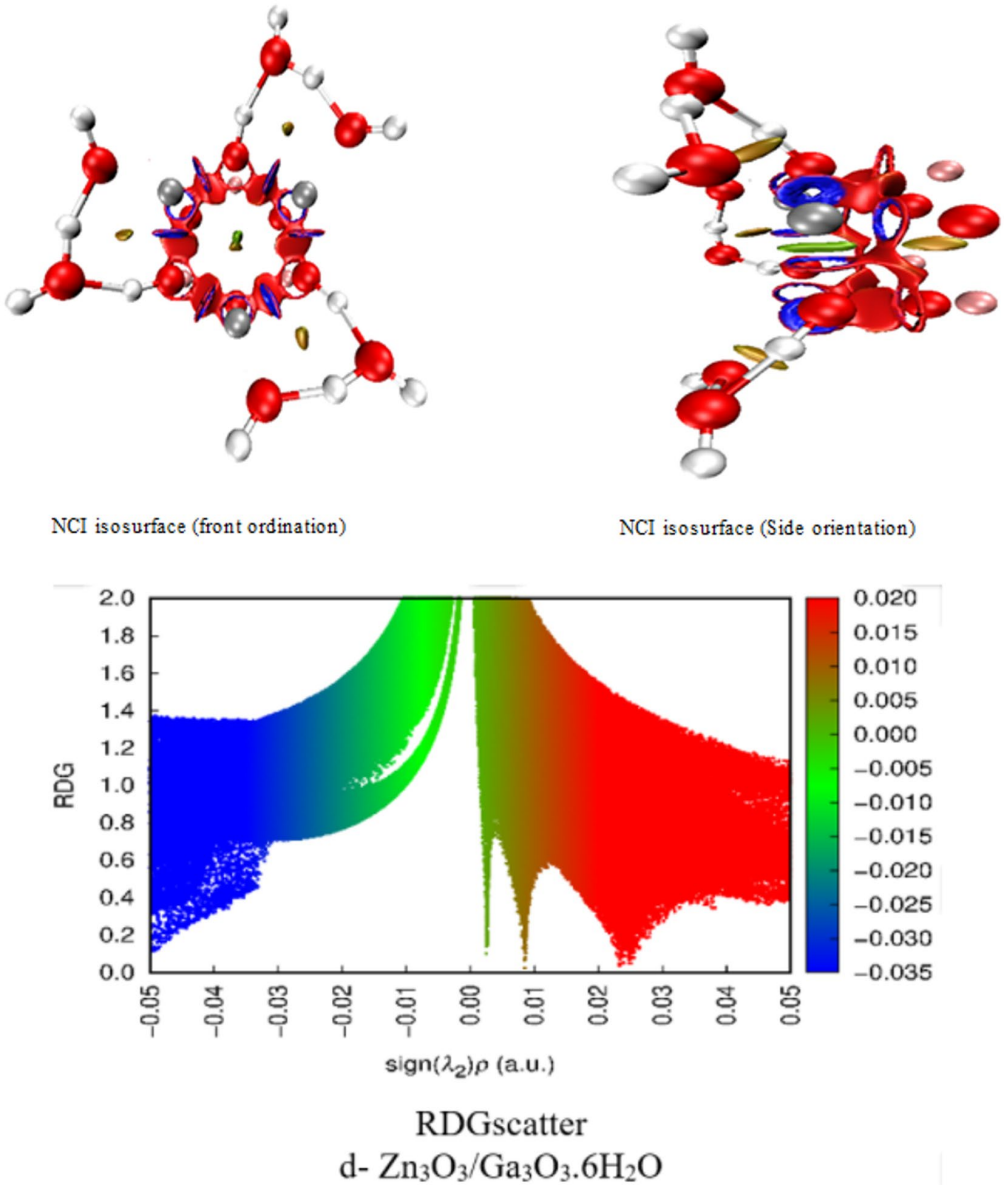
Visualization of noncovalent interactions in the for (**a**) Zn_3_O_3_, (**b**) Ga_3_O_3_ (**c**) Zn_3_O_3_/Ga_3_O_3_ composite and (**d**) Zn_3_O_3_/Ga_3_O_3_ composite interacting with 6 water molecules. systems showing the NCI isosurface (left) and corresponding RDG scatter plot (right), colored by sign(λ₂)ρ, highlighting dominant steric effects and weak dispersive interactions within the Zn–O (Ga–O ) framework. Blue, green, and red regions represent strong attractive, weak van der Waals, and steric repulsive interactions, respectively.

The noncovalent interaction (NCI) analysis provides a comprehensive visualization of the intermolecular forces governing water adsorption behavior across the composite systems. For the pristine Zn₃O₃ cluster (Figure 6a), the NCI isosurface reveals localized regions of weak van der Waals interactions (yellow–green) between oxygen and zinc atoms within the ring structure, along with small steric repulsion zones (red) near the metal centers, while the reduced density gradient (RDG) scatter plot displays characteristic spikes in the low-density, low-gradient region (sign(λ₂)ρ ≈ − 0.02 to 0.00 a.u.), indicating predominantly weak noncovalent interactions with limited spatial extent. The Ga₃O₃ cluster (Figure 6b) exhibits a similar pattern but with slightly more diffuse yellow–green isosurfaces around the Ga–O framework and broader distribution in the RDG scatter plot, suggesting enhanced polarizability and more extensive van der Waals interaction networks compared to Zn₃O₃. The mixed oxide composite Zn₃O₃/Ga₃O₃ (Figure 6c) demonstrates a dramatic transformation in the NCI landscape, characterized by extensive blue isosurfaces that span across the entire interfacial region between the two oxide domains, coupled with multiple red regions indicating controlled steric repulsion at specific interaction sites. The corresponding RDG scatter plot reveals significantly expanded features across a broader range of sign(λ₂)ρ values (− 0.04 to 0.02 a.u.), with intensified blue regions indicating the formation of numerous moderate-to-strong attractive interactions that are absent in the individual pristine clusters. Upon hydration, the Zn₃O₃/Ga₃O₃·6 H₂O system (Figure 6d) displays a remarkably complex NCI topology with pervasive blue isosurfaces enveloping the water molecules and bridging them to the oxide framework, indicating strong hydrogen-bonding networks (sign(λ₂)ρ < − 0.03 a.u.), while green isosurfaces fill the interstitial spaces between water molecules and the composite surface, representing stabilizing van der Waals contributions. The RDG scatter plot for the hydrated system shows distinct blue spikes at highly negative sign(λ₂)ρ values characteristic of O–H···O hydrogen bonds, alongside broadened green features corresponding to dispersive water-oxide and water-water interactions. From a composite design perspective, the NCI analysis reveals that the heterogeneous Zn₃O₃/Ga₃O₃ interface generates spatially extended interaction domains that are critical for water accommodation. The integration of distinct metal–oxygen frameworks creates complementary binding pockets in which attractive van der Waals interactions (green) dominate, accompanied by pronounced hydrogen-bonding regions (blue). Compared to the localized and relatively weak interactions observed in the pristine clusters, the composite system exhibits extensive, multi-centered noncovalent interaction networks, demonstrating that interfacial engineering between Zn₃O₃ and Ga₃O₃ significantly enhances the overall binding capacity. Furthermore, the predominance of blue isosurfaces in the hydrated composite confirms the formation of strong yet noncovalent water–surface interactions mediated by cooperative hydrogen bonding, where individual water molecules simultaneously interact with surface oxygen sites and neighboring water molecules. This cooperative effect maximizes adsorption capacity while preserving reversibility. The balanced distribution of attractive blue–green NCI isosurfaces validates that the Zn₃O₃/Ga₃O₃ composite architecture optimizes both geometric and electronic complementarity, generating multiple energetically favorable adsorption sites that collectively enhance hygroscopic performance beyond that achievable by either oxide component alone.

### QTAIM analysis

#### Nuclear critical point (NCP) analysis

**Fig. 7:**
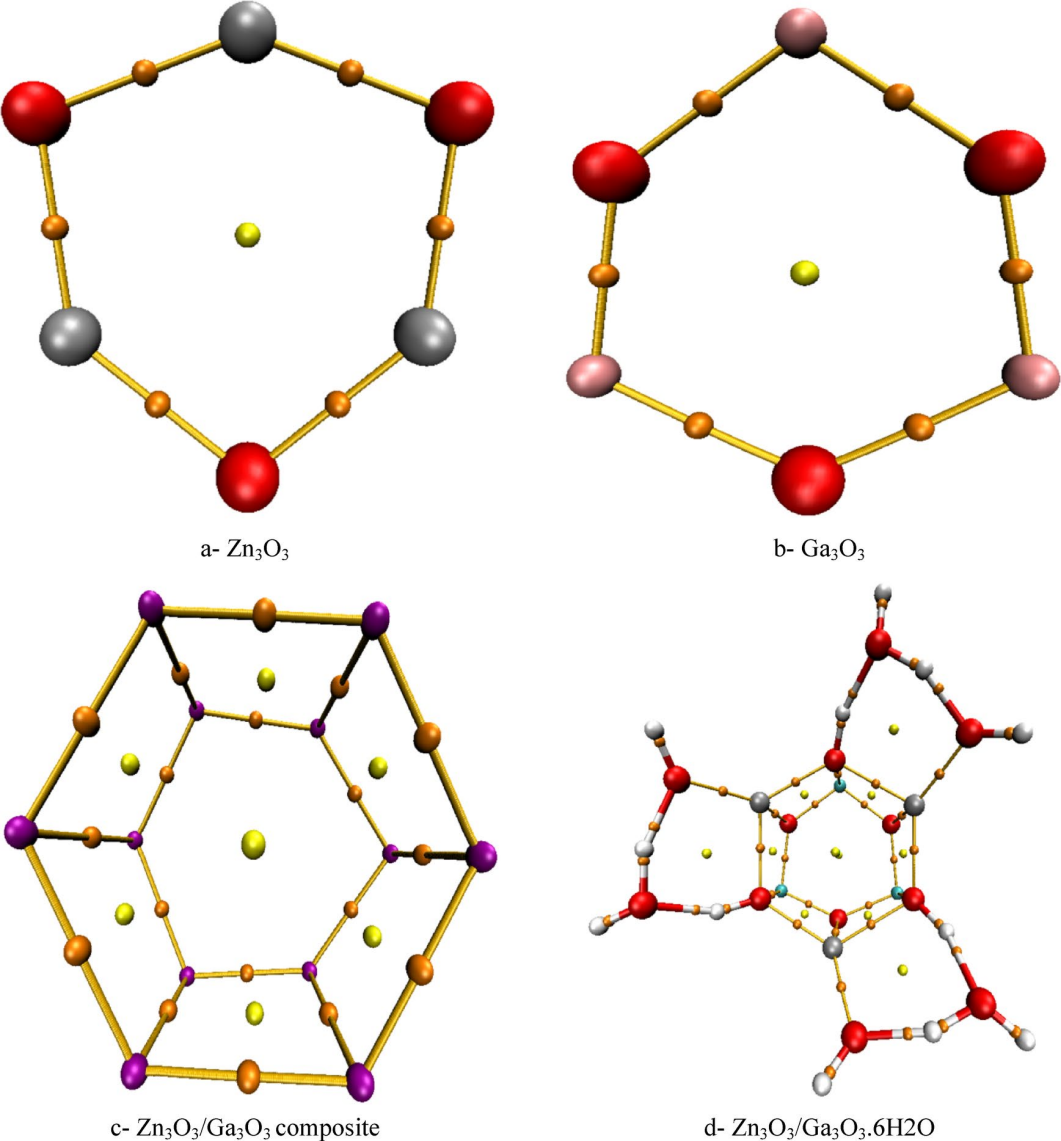
Visualization of QTAIM analysis of for (**a**) Zn_3_O_3_, (**b**) Ga_3_O_3_ (**c**) Zn_3_O_3_/Ga_3_O_3_ composite and (**d**) Zn_3_O_3_/Ga_3_O_3_ composite interacting with 6 water molecules.

The QTAIM analysis of the Zn₃O₃ cluster (Figure 7a; Table 3) reveals pronounced electron localization at the atomic nuclei, as reflected by the very high electron density values at the nuclear critical points (ρ = 35106.89 a.u.) and strongly negative Laplacian values (∇²ρ = −7.17 × 10¹⁰ a.u.) associated with the zinc centers. In QTAIM theory, such large electron density and negative Laplacian values at NCPs primarily arise from core-electron concentration and therefore describe atomic identity and electronic localization rather than chemical bonding interactions. Oxygen atoms exhibit substantially lower electron densities (ρ ≈ 295.23 a.u.) with moderately negative Laplacian values (∇²ρ ≈ −2.35 × 10⁶ a.u.), consistent with their lighter atomic character and reduced core-electron density.

The Average Local Ionization Energy (ALIE) distribution shows clear differentiation between atomic species, with zinc atoms displaying lower ALIE values (0.60 a.u.) compared with oxygen atoms (18.19 a.u.). Because ALIE evaluated at nuclear regions is dominated by atomic ionization characteristics, these values are interpreted qualitatively as indicators of relative electronic softness rather than direct measures of bonding strength or adsorption energy. The uniformity of QTAIM and ALIE descriptors across equivalent zinc and oxygen atoms indicates a highly symmetric electronic structure, suggesting comparable atomic electronic environments rather than differences in bonding interactions.

For the Ga₃O₃ cluster (Figure 7b; Table 4), a similar electronic topology is observed. Gallium atoms present very high electron densities at NCPs (ρ = 36588.52 a.u.) with strongly negative Laplacian values (∇²ρ = −5.65 × 10¹⁰ a.u.), again reflecting dominant core-electron localization at the metal nuclei. Oxygen atoms retain electron densities comparable to those observed in Zn₃O₃ (ρ = 295.12–295.45 a.u.). Gallium atoms exhibit slightly lower ALIE values (0.41–0.43 a.u.) relative to zinc, indicating comparatively softer electronic character, while oxygen atoms maintain consistently high ALIE values (≈ 18.22 a.u.). Minor variations in ALIE among gallium atoms suggest small electronic asymmetries within the cluster, which may contribute to differentiated interaction environments without implying direct bonding differences at the nuclear level.

The Zn₃O₃/Ga₃O₃ heterostructure (Figure 7c; Table 5) exhibits a more complex electronic distribution characterized by twelve nuclear critical points corresponding to the combined framework. Metal centers preserve their characteristic high NCP electron densities (Zn: 35106.89 a.u.; Ga: 36588.52 a.u.), while oxygen atoms display consistent intermediate values (295.12–295.37 a.u.). Changes in ALIE values are observed upon heterostructure formation, with zinc sites showing slightly increased ALIE values (0.78–0.79 a.u.), suggesting redistribution of electronic density within the combined system. Gallium atoms retain comparatively low ALIE values (0.38–0.43 a.u.), whereas oxygen atoms maintain high values (18.20–18.21 a.u.). These variations indicate electronic reorganization associated with heterostructure formation, reflecting differences in local electronic environments rather than direct evidence of bonding interactions.

Upon hydration (Zn₃O₃/Ga₃O₃·6 H₂O, Figure 7d; Table 6), the nuclear critical point properties remain dominated by strong metal-centered electron localization, indicating preservation of the intrinsic electronic framework. Variations in ALIE values are observed following water coordination, with zinc atoms showing reduced ALIE values (~ 0.55 a.u.), consistent with electronic redistribution induced by interaction with water molecules, while gallium atoms maintain relatively stable values (~ 0.41 a.u.). Framework oxygen atoms exhibit only minor changes in electron density (294.63–294.99 a.u.) and retain high ALIE values (18.21–18.26 a.u.). Newly introduced NCPs associated with water molecules display characteristic atomic electron density values corresponding to hydrogen and oxygen nuclei, reflecting incorporation of water into the electronic environment of the system.

Overall, NCP analysis confirms preservation of atomic electronic localization and structural integrity across all systems. Because nuclear critical point properties primarily reflect core-electron behavior, detailed characterization of adsorption and intermolecular interactions is discussed based on bond critical point (BCP) analysis in the following section.

**Table 3:** Quantum Theory of Atoms in Molecules (QTAIM) analysis of Zn_3_O_3 _at nuclear critical points (NCP), presenting electron density ρ (a.u.), its Laplacian ∇²ρ (a.u.), and Average Local Ionization Energy (ALIE) (a.u.) for each atomic nucleus, which characterize the molecular electron distribution and site-specific ionization properties.

CP	Corresponding nucleus	Electron density ρ (a.u.)	Laplacian ∇²ρ (a.u.)	ALIE (a.u.)
1	3(O)	295.23	− 2352032.87	18.19
2	5(Zn)	35106.89	− 71668833840.00	0.60
3	6(Zn)	35106.89	− 71668833840.00	0.60
4	2(O)	295.23	− 2352032.83	18.19
5	1(O)	295.23	− 2352032.83	18.19
6	4(Zn)	35106.89	− 71668833840.00	0.60

**Table 4:** Quantum Theory of Atoms in Molecules (QTAIM) analysis of Ga_3_O_3_ at nuclear critical points (NCP), presenting electron density ρ (a.u.), its Laplacian ∇²ρ (a.u.), and Average Local Ionization Energy (ALIE) (a.u.) for each atomic nucleus, which characterize the molecular electron distribution and site-specific ionization properties.

CP	Corresponding nucleus	Electron density ρ (a.u.)	Laplacian ∇²ρ (a.u.)	ALIE (a.u.)
1	6(O)	295.45	− 2353919.73	18.22
2	2(Ga)	36588.52	− 56499274370.00	0.43
3	3(Ga)	36588.52	− 56499274370.00	0.43
4	5(O)	295.12	− 2351129.81	18.22
5	4(O)	295.12	− 2351129.81	18.22
6	1(Ga)	36588.52	− 56499274370.00	0.41

**Table 5:** Quantum Theory of Atoms in Molecules (QTAIM) analysis of Zn_3_O_3_/Ga_3_O_3 _at nuclear critical points (NCP), presenting electron density ρ (a.u.), its Laplacian ∇²ρ (a.u.), and Average Local Ionization Energy (ALIE) (a.u.) for each atomic nucleus, which characterize the molecular electron distribution and site-specific ionization properties.

CP	Corresponding nucleus	Electron density ρ (a.u.)	Laplacian ∇²ρ (a.u.)	ALIE (a.u.)
1	12(O)	295.12	− 2351203.39	18.21
2	9(Ga)	36588.52	− 56499274370.00	0.43
3	5(Zn)	35106.89	− 71668833840.00	0.79
4	3(O)	295.28	− 2352568.48	18.21
5	8(Ga)	36588.52	− 56499274370.00	0.38
6	10(O)	295.37	− 2353326.74	18.21
7	2(O)	295.20	− 2351893.71	18.20
8	6(Zn)	35106.89	− 71668833840.00	0.78
9	11(O)	295.12	− 2351204.60	18.21
10	7(Ga)	36588.52	− 56499274370.00	0.43
11	4(Zn)	35106.89	− 71668833840.00	0.79
12	1(O)	295.28	− 2352568.18	18.21

**Table 6:** Quantum Theory of Atoms in Molecules (QTAIM) analysis of Zn_3_O_3_/Ga_3_O_3_.6H_2_O at nuclear critical points (NCP), presenting electron density ρ (a.u.), its Laplacian ∇²ρ (a.u.), and Average Local Ionization Energy (ALIE) (a.u.) for each atomic nucleus, which characterize the molecular electron distribution and site-specific ionization properties.

CP	Corresponding nucleus	Electron Density ρ (a.u.)	Laplacian ∇²ρ (a.u.)	ALIE (a.u.)
1	16(H)	0.41	− 17.83	0.61
2	15(O)	294.68	− 2347350.48	18.23
3	20(H)	0.32	− 14.09	0.60
4	25(H)	0.40	− 17.65	0.63
5	27(H)	0.40	− 17.65	0.63
6	26(O)	294.63	− 2346878.98	18.26
7	24(O)	294.63	− 2346881.22	18.26
8	5(Zn)	35106.89	− 71668833840.00	0.55
9	30(H)	0.32	− 14.13	0.61
10	3(O)	294.80	− 2348479.19	18.26
11	14(H)	0.32	− 14.10	0.60
12	28(H)	0.32	− 14.11	0.61
13	12(O)	294.91	− 2349397.80	18.22
14	9(Ga)	36588.52	− 56499274370.00	0.41
15	2(O)	294.80	− 2348476.94	18.26
16	8(Ga)	36588.52	− 56499274370.00	0.41
17	13(O)	294.68	− 2347344.21	18.23
18	6(Zn)	35106.89	− 71668833840.00	0.55
19	19(H)	0.41	− 17.83	0.61
20	10(O)	294.99	− 2350108.02	18.21
21	4(Zn)	35106.89	− 71668833840.00	0.55
22	11(O)	294.99	− 2350090.21	18.21
23	1(O)	294.81	− 2348544.68	18.26
24	21(H)	0.41	− 17.82	0.61
25	17(O)	294.68	− 2347337.81	18.24
26	7(Ga)	36588.52	− 56499274370.00	0.41
27	29(H)	0.32	− 14.08	0.61
28	18(H)	0.32	− 14.07	0.60
29	22(O)	294.63	− 2346867.27	18.26
30	23(H)	0.40	− 17.65	0.63

### Bond critical point (BCP) analysis

Bond types were assigned according to established QTAIM criteria based on the combined evaluation of electron density, Laplacian, and total energy density, distinguishing shared-shell (covalent) from closed-shell interactions (hydrogen bonding and metal–ligand coordination). Topological analysis reveals highly uniform Zn–O coordination across the Zn₃O₃ framework (Figure 7a; Table 7), with identical electron density at the bond critical points (ρ = 0.10 a.u.), consistent kinetic energy density (G = 0.13 a.u.), potential energy density (V = − 0.15 a.u.), and slightly negative total energy density (H = − 0.02 a.u.). The uniformly positive Laplacian (∇²ρ = 0.57 a.u.) and minimal ellipticity (ε = 0.02) indicate predominantly closed-shell metal–oxygen coordination interactions with a limited degree of covalent contribution. While this structural symmetry ensures equivalent zinc reactivity and predictable adsorption behavior, the moderate electron density suggests intermediate interaction strength, providing structural stability but reduced electronic flexibility for dynamic water coordination. The rigid, low-ellipticity bonding environment may therefore restrict the formation of extended hydrogen-bonding networks, potentially limiting water uptake capacity despite favorable structural uniformity.

In contrast, the Ga₃O₃ framework exhibits bonding heterogeneity (Figure 7b; Table 8), with two distinct Ga–O bond classes. Four bonds show moderate electron density (ρ = 0.12 a.u.) and lower kinetic energy density, whereas two bonds display slightly higher electron density (ρ = 0.13 a.u.) accompanied by more negative potential and total energy densities. All Ga–O interactions maintain low ellipticity (ε ≈ 0.01) and negative H(r) values (− 0.03 to − 0.04 a.u.), confirming stable closed-shell coordination interactions with enhanced covalent polarization compared to Zn–O bonds. This differentiation generates chemically distinct sites, where stronger Ga–O bonds impart framework rigidity and thermal stability, while relatively weaker bonds provide more labile coordination environments favorable for water adsorption. The overall higher electron density compared with Zn₃O₃ correlates with lower ALIE values at gallium centers, indicating enhanced Lewis acidity and a stronger affinity for water molecules, supporting improved intrinsic water-binding capability.

The heterostructure Zn₃O₃/Ga₃O₃ displays a hierarchical bonding landscape spanning strong, moderate, and weak interactions (Figure 7c; Table 9) (ρ = 0.05–0.11 a.u.), reflecting interfacial complexity absent in the pristine oxides. Strong Ga–O bonds (ρ = 0.10–0.11 a.u., H ≈ − 0.03 a.u.) provide structural anchoring, whereas moderate Zn–O and mixed metal–oxygen interactions ensure framework connectivity. Critically, weak interfacial bonds (ρ = 0.05–0.06 a.u., H ≈ − 0.01 a.u.) introduce electronically flexible, low-energy interaction sites capable of accommodating incoming water molecules. The coexistence of multiple bond strengths creates a gradient of water affinity, enabling high-affinity adsorption at gallium-rich regions, intermediate binding at zinc sites, and facilitated diffusion through weak interfacial domains. This synergistic bonding hierarchy is particularly advantageous for simultaneously enhancing adsorption capacity and adsorption kinetics while maintaining structural robustness.

Upon hydration, the system (Zn₃O₃/Ga₃O₃·6 H₂O, Figure 7d; Table 10) develops an extensive and diverse bonding network comprising covalent O–H bonds within water molecules, classical hydrogen bonds, metal–oxygen coordination interactions and preserved internal framework bonds. Strong covalent O–H bonds exhibit high electron density (ρ = 0.25–0.33 a.u.) and strongly negative H(r), while hydrogen bonds display moderate density (ρ = 0.07–0.08 a.u.), consistent with cooperative water–water and water–framework interactions. Direct coordination of water molecules to metal centers (ρ ≈ 0.09 a.u.) confirms zinc sites as primary adsorption anchors, whereas selected water–framework interactions exhibit covalent-like topological characteristics (ρ ≈ 0.26 a.u.), suggesting strong hydrogen bonding or partial proton sharing rather than true covalent bond formation. Importantly, the framework retains its structural integrity upon hydration, with internal bond densities remaining largely preserved.

These results indicate a multilevel adsorption mechanism in which initial chemisorption at metal sites is reinforced by extensive hydrogen-bonding networks, enabling multilayer water uptake. The coexistence of strong anchoring interactions and weaker, flexible bonds supports high water capacity while allowing structural relaxation necessary for reversible adsorption–desorption cycling. Collectively, the heterostructure achieves an optimal balance between binding strength, uptake capacity, and regeneration efficiency, highlighting its potential suitability for atmospheric water harvesting applications.

**Table 7:** QTAIM topological and energetic parameters at bond critical points (BCP) for (Zn_3_O_3_**)**, presenting interatomic connections, electron density ρ(r) (a.u.), kinetic energy density G(r) (a.u.), potential energy density V(r) (a.u.), total energy density H(r) (a.u.), Laplacian ∇²ρ(r) (a.u.), ellipticity ε, and bonding classification.

CP	Connected atoms	ρ(*r*)	G(*r*)	V(*r*)	H(*r*)	∇²ρ(*r*)	ε	Bond type
7	3(O)–5(Zn)	0.10	0.13	− 0.15	− 0.02	0.57	0.02	Metal–oxygen coordination bond
8	3(O)–6(Zn)	0.10	0.13	− 0.15	− 0.02	0.57	0.02	Metal–oxygen coordination bond
9	5(Zn)–2(O)	0.10	0.13	− 0.15	− 0.02	0.57	0.02	Metal–oxygen coordination bond
11	6(Zn)–1(O)	0.10	0.13	− 0.15	− 0.02	0.57	0.02	Metal–oxygen coordination bond
12	2(O)–4(Zn)	0.10	0.13	− 0.15	− 0.02	0.57	0.02	Metal–oxygen coordination bond
13	1(O)–4(Zn)	0.10	0.13	− 0.15	− 0.02	0.57	0.02	Metal–oxygen coordination bond

**Table 8:** QTAIM topological and energetic parameters at bond critical points (BCP) for (Ga_3_O_3_**)**, presenting interatomic connections, electron density ρ(r) (a.u.), kinetic energy density G(r) (a.u.), potential energy density V(r) (a.u.), total energy density H(r) (a.u.), Laplacian ∇²ρ(r) (a.u.), ellipticity ε, and bonding classification.

CP	Connected atoms	ρ(*r*)	G(*r*)	V(*r*)	H(*r*)	∇²ρ(*r*)	ε	Bond type
7	6(O)–2(Ga)	0.12	0.06	− 0.10	− 0.03	0.62	0.01	Metal–oxygen coordination bond
8	6(O)–3(Ga)	0.12	0.06	− 0.10	− 0.03	0.62	0.01	Metal–oxygen coordination bond
9	2(Ga)–5(O)	0.13	0.08	− 0.11	− 0.04	0.74	0.01	Metal–oxygen coordination bond
11	3(Ga)–4(O)	0.13	0.08	− 0.11	− 0.04	0.74	0.01	Metal–oxygen coordination bond
12	5(O)–1(Ga)	0.12	0.06	− 0.09	− 0.03	0.62	0.01	Metal–oxygen coordination bond
13	4(O)–1(Ga)	0.12	0.06	− 0.09	− 0.03	0.62	0.01	Metal–oxygen coordination bond

**Table 9:** QTAIM topological and energetic parameters at bond critical points (BCP) for (Zn_3_O_3_/Ga_3_O_3_**)**, presenting interatomic connections, electron density ρ(r) (a.u.), kinetic energy density G(r) (a.u.), potential energy density V(r) (a.u.), total energy density H(r) (a.u.), Laplacian ∇²ρ(r) (a.u.), ellipticity ε, and bonding classification.

CP	Connected atoms	ρ(*r*)	G(*r*)	V(*r*)	H(*r*)	∇²ρ(*r*)	ε	Bond type
13	12(O)–9(Ga)	0.11	0.06	− 0.09	− 0.03	0.58	0.03	Metal–oxygen coordination bond
14	12(O)–5(Zn)	0.06	0.07	− 0.08	− 0.01	0.27	0.02	weak (metal–oxygen coordination bond)
15	9(Ga)–3(O)	0.09	0.04	− 0.07	− 0.02	0.42	0.04	Moderate (metal–oxygen coordination bond)
17	5(Zn)–3(O)	0.08	0.09	− 0.11	− 0.02	0.41	0.04	Moderate (metal–oxygen coordination bond)
18	12(O)–8(Ga)	0.09	0.05	− 0.07	− 0.02	0.45	0.03	Moderate (metal–oxygen coordination bond)
19	9(Ga)–10(O)	0.10	0.05	− 0.08	− 0.03	0.51	0.03	Metal–oxygen coordination bond
21	5(Zn)–2(O)	0.08	0.10	− 0.12	− 0.02	0.46	0.03	Moderate (metal–oxygen coordination bond)
23	3(O)–6(Zn)	0.08	0.09	− 0.11	− 0.02	0.41	0.04	Moderate (metal–oxygen coordination bond)
25	8(Ga)–2(O)	0.08	0.03	− 0.05	− 0.02	0.34	0.04	Moderate (metal–oxygen coordination bond)
26	10(O)–6(Zn)	0.05	0.05	− 0.06	− 0.01	0.19	0.01	weak (metal–oxygen coordination bond)
27	8(Ga)–11(O)	0.09	0.05	− 0.07	− 0.02	0.45	0.03	Moderate (metal–oxygen coordination bond)
30	10(O)–7(Ga)	0.10	0.05	− 0.08	− 0.03	0.51	0.03	Metal–oxygen coordination bond
32	2(O)–4(Zn)	0.08	0.10	− 0.12	− 0.02	0.45	0.03	Moderate (metal–oxygen coordination bond)
33	6(Zn)–1(O)	0.08	0.09	− 0.11	− 0.02	0.41	0.04	Moderate (metal–oxygen coordination bond)
34	11(O)–7(Ga)	0.11	0.06	− 0.09	− 0.03	0.58	0.03	Metal–oxygen coordination bond
35	11(O)–4(Zn)	0.06	0.07	− 0.08	− 0.01	0.27	0.02	Weak (metal–oxygen coordination bond)
37	7(Ga)–1(O)	0.09	0.04	− 0.07	− 0.02	0.42	0.04	Moderate (metal–oxygen coordination bond)
38	4(Zn)–1(O)	0.08	0.09	− 0.11	− 0.02	0.41	0.04	Moderate (metal–oxygen coordination bond)

**Table 10:** QTAIM topological and energetic parameters at bond critical points (BCP) for (Zn_3_O_3_/Ga_3_O_3_.6H_2_O**)**, presenting interatomic connections, electron density ρ(r) (a.u.), kinetic energy density G(r) (a.u.), potential energy density V(r) (a.u.), total energy density H(r) (a.u.), Laplacian ∇²ρ(r) (a.u.), ellipticity ε, and bonding classification.

CP	Connected atoms	ρ(*r*)	G(*r*)	V(*r*)	H(*r*)	∇²ρ(*r*)	ε	Bond type
31	16(H)–15(O)	0.33	0.07	− 0.50	− 0.43	− 1.44	0.01	Covalent bond (inside the water molecules)
32	15(O)–20(H)	0.08	0.07	− 0.09	− 0.02	0.18	0.03	H-bond (inside the water molecules)
33	20(H)–26(O)	0.26	0.07	− 0.39	− 0.32	− 1.02	0.02	Covalent bond (inside the water molecules)
34	15(O)–5(Zn)	0.09	0.12	− 0.14	− 0.02	0.56	0.05	Metal–oxygen coordination bond (between the absorbent and water molecule)
35	25(H)–24(O)	0.33	0.07	− 0.50	− 0.43	− 1.46	0.02	Covalent bond (inside the water molecules)
36	27(H)–26(O)	0.33	0.07	− 0.50	− 0.43	− 1.46	0.02	Covalent bond (inside the water molecules)
38	24(O)–30(H )	0.07	0.06	− 0.08	− 0.02	0.19	0.04	H-bond (inside the water molecules)
39	30(H)–3(O)	0.26	0.07	− 0.39	− 0.33	− 1.04	0.00	Strong hydrogen bond between water hydrogen and adsorbent oxygen
40	5(Zn)–3(O)	0.07	0.08	− 0.09	− 0.01	0.35	0.02	Metal–oxygen coordination bond (inside the absorbent)
41	5(Zn)–12(O)	0.07	0.08	− 0.09	− 0.01	0.33	0.03	Metal–oxygen coordination bond (inside the absorbent)
42	26(O)–28(H)	0.07	0.06	− 0.08	− 0.02	0.19	0.04	H-bond (inside the water molecules)
43	24(O)–14(H)	0.26	0.07	− 0.39	− 0.32	− 1.02	0.02	Covalent bond (inside the water molecules)
45	5(Zn)–2(O)	0.05	0.06	− 0.07	− 0.01	0.23	0.03	Weak (metal–oxygen coordination bond) (inside absorbent)
46	3(O)–9(Ga)	0.07	0.03	− 0.05	− 0.01	0.35	0.01	Moderate (metal–oxygen coordination bond) (inside the molecular frame)
47	28(H)–2(O)	0.26	0.07	− 0.39	− 0.33	− 1.04	0.00	Strong hydrogen bond between water hydrogen and adsorbent oxygen
49	12(O)–9(Ga)	0.11	0.06	− 0.08	− 0.03	0.56	0.03	Metal–oxygen coordination bond (inside the absorbent)
51	14(H)–13(O)	0.08	0.07	− 0.09	− 0.02	0.19	0.03	H-bond (inside the water molecules)
52	12(O)–8(Ga)	0.11	0.06	− 0.08	− 0.03	0.56	0.03	Metal–oxygen coordination bond (inside the absorbent)
53	3(O)–6(Zn)	0.05	0.06	− 0.07	− 0.01	0.24	0.03	Metal–oxygen coordination bond (inside the absorbent)
54	2(O)–8(Ga)	0.07	0.03	− 0.05	− 0.01	0.35	0.02	Metal–oxygen coordination bond (inside the absorbent)
57	9(Ga)–10(O)	0.11	0.05	− 0.08	− 0.03	0.54	0.03	Metal–oxygen coordination bond (inside the absorbent)
58	2(O)–4(Zn)	0.07	0.08	− 0.09	− 0.01	0.35	0.02	Metal–oxygen coordination bond (inside the absorbent)
59	13(O)–6(Zn)	0.09	0.12	− 0.14	− 0.02	0.56	0.05	Metal–oxygen coordination bond (between the absorbent and water molecule)
62	13(O)–19(H)	0.33	0.07	− 0.50	− 0.43	− 1.44	0.01	Covalent (inside water molecule)
63	8(Ga)–11(O)	0.11	0.05	− 0.08	− 0.03	0.53	0.03	Metal–oxygen coordination bond (inside the absorbent)
64	6(Zn)–10(O)	0.06	0.07	− 0.09	− 0.01	0.31	0.03	Metal–oxygen coordination bond (inside the absorbent)
65	6(Zn)–1(O)	0.07	0.08	− 0.09	− 0.01	0.35	0.02	Metal–oxygen coordination bond (inside the absorbent)
67	4(Zn)–11(O)	0.06	0.08	− 0.09	− 0.01	0.32	0.03	Metal–oxygen coordination bond (inside the absorbent)
68	4(Zn)–1(O)	0.05	0.06	− 0.07	− 0.01	0.23	0.03	Metal–oxygen coordination bond (inside the absorbent)
69	10(O)–7(Ga)	0.11	0.05	− 0.08	− 0.03	0.55	0.03	Metal–oxygen coordination bond (inside the absorbent)
71	4(Zn)–17(O)	0.09	0.12	− 0.14	− 0.02	0.56	0.05	Metal–oxygen coordination bond (between the absorbent and water molecule)
72	11(O)–7(Ga)	0.11	0.06	− 0.08	− 0.03	0.55	0.03	Metal–oxygen coordination bond (inside the absorbent)
73	21(H)–17(O)	0.33	0.07	− 0.50	− 0.43	− 1.44	0.01	Covalent (inside water molecule)
74	1(O)–7(Ga)	0.07	0.04	− 0.05	− 0.01	0.35	0.02	Metal–oxygen coordination bond (inside the absorbent)
76	1(O)–29(H)	0.26	0.07	− 0.39	− 0.32	− 1.03	0.00	Strong hydrogen bond between adsorbent oxygen and water hydrogen
77	17(O)–18(H)	0.08	0.07	− 0.09	− 0.02	0.18	0.03	H-bond (inside the water molecules)
78	29(H)–22(O)	0.08	0.06	− 0.08	− 0.02	0.19	0.04	H-bond (inside the water molecules)
79	18(H)–22(O)	0.25	0.07	− 0.39	− 0.32	− 1.02	0.02	Covalent (inside water molecule)
80	22(O)–23(H)	0.33	0.07	− 0.50	− 0.43	− 1.46	0.02	Covalent (inside water molecule)

### Natural bond orbital (NBO) analysis of Zn_3_O_3_, Ga_3_O_3_, Zn_3_O_3_^/^Ga_3_O_3_, and water adsorption

To elucidate the nature of intramolecular interactions and interfacial charge transfer in the studied systems, second-order perturbation theory analysis within the NBO framework was performed. In the pristine Zn_3_O_3_ ring (Table 11), the dominant stabilizing interactions arise from delocalization of bonding electron density between symmetry-equivalent Zn–O bonds, with BD(O–Zn) → BD*(O–Zn) interactions uniformly yielding a second-order stabilization energy E(2) of 10.39 kcal/mol for all six symmetry-related donor–acceptor pairs. Concurrently, the oxygen lone pairs LP(2) on each O atom donate into the formally vacant LP*(6) orbitals on the adjacent Zn centers, contributing an additional 10.20 kcal/mol per interaction, with relatively modest off-diagonal Fock matrix elements F(i, j) of 0.047 a.u. and energy gaps E(j)–E(i) of 0.27 a.u. The uniformity and moderate magnitude of these interactions reflect the symmetric, homoleptic character of the 3ZnO six-membered ring, indicative of a predominantly ionic bonding framework stabilized by equivalent Zn–O σ-type hyperconjugative delocalization.

In stark contrast, the Ga_3_O_3_ ring (Table 12) exhibits markedly richer and stronger NBO interactions, consistent with the greater covalent character of Ga–O bonding and the presence of low-lying empty Ga-based acceptor orbitals. The most striking interaction is LP*(1)Ga3 → BD*(1)Ga1–O4, with an exceptionally large E(2) of 158.52 kcal/mol, accompanied by a vanishingly small energy gap of only 0.05 a.u. and an F(i, j) of 0.270 a.u., signaling near-resonant charge transfer between Ga centers mediated through the ring framework. LP(3)O4 → BD*(1)Ga1–O4 contributes 38.87 kcal/mol, while LP(4)O6 → BD(1)Ga2–Ga3 yields 40.68 kcal/mol, highlighting the participation of oxygen lone pairs in stabilizing Ga–Ga interactions. Additional strong contributions include BD*(1)Ga2–Ga3 → BD*(1)Ga2–O5 (35.97 kcal/mol), LP*(1)Ga2 → BD*(1)Ga2–O5 (53.58 kcal/mol), and BD(1)Ga1–O4 → LP*(1)Ga3 (33.65 kcal/mol), all underscoring the extensive electron delocalization and the critical role of gallium empty orbitals as electron acceptors. These interactions collectively indicate a highly polarized and electronically flexible Ga_3_O_3_ ring, primed for interfacial charge transfer upon heterostructure formation.

Upon assembly of the Zn_3_O_3_^/^Ga_3_O_3_ heterostructure (Table 13), the NBO analysis reveals a pronounced interfacial electronic coupling between the two component rings. The oxygen lone pairs LP(2)O1 on the ZnO subunit donate into the empty Ga-based LP*(1)Ga7 orbital (E(2) = 10.92 kcal/mol, F(i, j) = 0.140 a.u.) and into the interfacial BD*(1)O1–Ga7 antibond (9.99 kcal/mol), while LP(2)O2 and LP(2)O3 on the ZnO ring engage Ga8 and Ga9 acceptor orbitals with E(2) values of 15.36 and 10.48 kcal/mol, respectively, through LP*(2,3)Ga interactions. Reciprocally, the Ga-based lone pairs LP(1)Ga9 donate back into BD*(1)Ga7–O10 (12.89 kcal/mol) and BD*(1)O3–Zn6 (15.37 kcal/mol), revealing a bidirectional charge-transfer mechanism at the Zn–O–Ga interface. Furthermore, BD(1)Zn5–O12 → LP*(2)Ga9 is particularly strong at 31.11 kcal/mol with F(i, j) = 0.195 a.u., and LP*(6)Zn5 → LP*(2)Ga9 contributes 20.41 kcal/mol, collectively demonstrating that the interfacial Zn and Ga centers act cooperatively as both electron donors and acceptors. The cross-ring BD*(1)O2–Zn4 → BD*(1)O1–Ga7 interaction (19.76 kcal/mol) further confirms that electron delocalization extends across the Zn_3_O_3_/Ga_3_O_3_ interface, giving rise to a covalently-coupled heterostructure with significant charge redistribution relative to the isolated rings.

The adsorption of six water molecules on the Zn_3_O_3_^/^Ga_3_O_3_ surface (Table 14) introduces a new layer of complexity, dominated by the coordinative interactions between water oxygen atoms and the Lewis-acidic Zn centers, as well as an extended hydrogen-bond network. The most prominent NBO interactions involve LP(3)O13 → LP*(6)Zn6 (21.95 kcal/mol) and LP(3)O17 → LP*(6)Zn4 (23.19 kcal/mol), and LP(3)O15 → LP*(6)Zn5 (24.92 kcal/mol), confirming strong dative coordination of the water lone pairs into the vacant d-type Zn orbitals, with associated F(i, j) values in the range 0.164–0.174 a.u. These interactions mirror classical Lewis acid–base coordination and are responsible for the strong chemisorptive affinity of the heterostructure surface toward water. In addition, the adsorbed water molecules engage in robust hydrogen-bonding interactions with the bridging oxygen atoms of the surface rings: LP(2)O17 → BD*(1)H18–O22 (29.39 kcal/mol), LP(2)O15 → BD*(1)H20–O26 (29.10 kcal/mol), and LP(2)O13 → BD*(1)H14–O24 (28.97 kcal/mol), all with F(i, j) ≈ 0.202–0.205 a.u., reflecting strong O–H···O hydrogen bonds that further stabilize the adsorbed water layer. Correspondingly, LP(2)O22 → BD*(1)O1–H29 (27.38 kcal/mol), LP(2)O24 → BD*(1)O3–H30 (26.77 kcal/mol), and LP(2)O26 → BD*(1)O2–H28 (26.94 kcal/mol) indicate that proton donors from the hydroxyl groups of adsorbed water interact with the surface oxygen atoms, establishing a cooperative hydrogen-bond relay between the surface and the water overlayer. In comparison, the isolated 6H_2_O ring (Table 15) displays symmetric LP(2)O → BD*(1)O–H hydrogen-bond interactions uniformly at 65.91–66.08 kcal/mol, values that are substantially quenched upon surface adsorption, consistent with the redistribution of water lone-pair density into Zn coordination bonds. The surface-induced attenuation of these intra-water hydrogen bonds, alongside the emergence of strong Zn–O water coordination, provides compelling NBO evidence that water adsorption on Zn_3_O_3_^/^Ga_3_O_3_ proceeds through a synergistic mechanism combining Lewis-acid coordination at Zn sites and hydrogen-bond stabilization at surface oxygen atoms, with the resulting adsorption configuration being thermodynamically robust and electronically well-coupled to the heterostructure framework. These NBO findings are in excellent agreement with the quantum theory of atoms in molecules (QTAIM) analysis, wherein the bond critical points, electron density ρ(r), and Laplacian ∇²ρ(r) values corroborate the same pattern of interfacial covalent-like coupling between Zn and Ga centers, the Lewis-acid coordinative character of the Zn–O (water) interactions, and the cooperative hydrogen-bond network stabilizing the adsorbed water overlayer on the Zn_3_O_3_Ga_3_O_3_ heterostructure surface.

**Table 11:** Second-order perturbation theory analysis of donor–acceptor interactions in the Natural Bond Orbital (NBO) framework for the Zn₃O₃ ring cluster.

Donor NBO (i)	Acceptor NBO (j)	E(2), kcal/mol	E(j) − E(i), a.u.	F(i, j), a.u.
BD (1) O 1–Zn 4	BD*(1) O 2–Zn 4	10.39	0.79	0.081
BD (1) O 1–Zn 6	BD*(1) O 3–Zn 6	10.39	0.79	0.081
BD (1) O 2–Zn 4	BD*(1) O 1–Zn 4	10.39	0.79	0.081
BD (1) O 2–Zn 5	BD*(1) O 3–Zn 5	10.39	0.79	0.081
BD (1) O 3–Zn 5	BD*(1) O 2–Zn 5	10.39	0.79	0.081
BD (1) O 3–Zn 6	BD*(1) O 1–Zn 6	10.39	0.79	0.081
LP (2) O 1	LP*(6)Zn 4	10.2	0.27	0.047
LP (2) O 1	LP*(6)Zn 6	10.2	0.27	0.047
LP (2) O 2	LP*(6)Zn 4	10.2	0.27	0.047
LP (2) O 2	LP*(6)Zn 5	10.2	0.27	0.047
LP (2) O 3	LP*(6)Zn 5	10.2	0.27	0.047
LP (2) O 3	LP*(6)Zn 6	10.2	0.27	0.047

**Table 12:** Second-order perturbation theory analysis of donor–acceptor interactions in the Natural Bond Orbital (NBO) framework for the Ga_3_O_3_ ring cluster.

Donor NBO (i)	Acceptor NBO (j)	E(2), kcal/mol	E(j) − E(i), a.u.	F(i, j), a.u.
LP (3) O 4	BD*(1)Ga 1–O 4	38.87	1.27	0.281
BD (1)Ga 1–O 4	LP*(1)Ga 3	33.65	0.88	0.219
LP*(2)Ga 1	BD*(1)Ga 2–O 5	28.21	0.19	0.209
LP*(1)Ga 3	BD*(1)Ga 1–O 4	158.52	0.05	0.27
LP (3) O 5	LP*(2)Ga 1	26.31	0.93	0.199
BD*(1)Ga 2–O 5	BD*(1)Ga 1–O 4	24.51	0.14	0.16
BD*(1)Ga 2–Ga 3	BD (1)Ga 2–Ga 3	31.39	0.07	0.063
BD*(1)Ga 2–Ga 3	BD*(1)Ga 2–O 5	35.97	0.59	0.188
LP*(1)Ga 2	BD*(1)Ga 2–O 5	53.58	0.08	0.187
LP*(1)Ga 3	RY*(3)Ga 3	23.36	12.89	2.248
BD*(1)Ga 2–O 5	RY*(2)Ga 2	24.06	1.07	0.551
BD*(1)Ga 2–O 5	RY*(3)Ga 2	23.23	12.45	1.855
LP (3) O 6	LP*(1)Ga 2	26.54	1.04	0.211
LP (4) O 6	BD (1)Ga 2–Ga 3	40.68	0.17	0.106

**Table 13:** Second-order perturbation theory analysis of donor–acceptor interactions in the Natural Bond Orbital (NBO) framework for the Zn_3_O_3_^/^Ga_3_O_3_ ring cluster.

Donor NBO (i)	Acceptor NBO (j)	E(2), kcal/mol	E(j) − E(i), a.u.	F(i, j), a.u.
LP (2) O 1	LP*(1)Ga 7	10.92	1.09	0.14
LP (2) O 1	BD*(1) O 1–Ga 7	9.99	0.89	0.123
BD*(1) O 1–Ga 7	LP*(1)Ga 7	9.83	0.2	0.112
LP (3) O 11	LP*(8)Zn 4	9.32	0.97	0.122
BD*(1) O 1–Ga 7	LP*(6)Zn 4	7.29	0.11	0.079
LP (2) O 10	LP*(6)Zn 6	7.47	0.54	0.08
LP (2) O 10	LP*(8)Zn 6	5.44	0.68	0.078
BD*(1)Ga 7–O 10	LP*(6)Zn 6	13.4	0.04	0.059
LP*(6)Zn 4	LP*(1)Ga 7	8.58	0.09	0.085
LP*(6)Zn 4	BD*(1)Ga 7–O 11	9.71	0.05	0.073
BD*(1) O 2–Zn 4	BD*(1) O 1–Ga 7	19.76	0.02	0.043
BD (1) O 2–Zn 4	BD*(1)Zn 5–O 12	7.42	0.28	0.062
LP (3) O 2	BD*(1)Zn 5–O 12	9.54	0.33	0.075
LP (2) O 2	LP*(2)Ga 8	15.36	1.07	0.166
LP (2) O 2	LP*(3)Ga 8	11.79	0.83	0.125
BD*(1) O 2–Zn 4	LP*(2)Ga 8	5.17	0.26	0.089
BD*(1) O 2–Zn 4	LP*(3)Ga 8	8.79	0.02	0.036
BD*(1) O 3–Zn 6	BD*(1) O 1–Ga 7	5.73	0.1	0.05
LP (3) O 3	BD*(1)Zn 5–O 12	8.53	0.27	0.064
BD (1) O 3–Zn 6	LP*(2)Ga 9	5.82	0.64	0.079
BD (1) O 3–Zn 6	LP*(3)Ga 9	6.17	0.42	0.064
LP (2) O 3	LP*(2)Ga 9	10.48	1.16	0.141
LP (2) O 3	LP*(3)Ga 9	11.06	0.93	0.128
BD*(1) O 3–Zn 6	LP*(3)Ga 9	6.77	0.14	0.071
BD*(1)Zn 5–O 12	BD*(1) O 2–Zn 4	14.24	0.09	0.079
BD*(1)Zn 5–O 12	BD*(1) O 3–Zn 6	7.91	0.01	0.021
LP (2) O 12	BD*(1)Zn 5–O 12	4.85	0.26	0.047
LP (3) O 12	LP*(8)Zn 5	9.11	0.92	0.118
LP (3) O 12	BD*(1)Zn 5–O 12	5.62	0.69	0.083
BD (1)Zn 5–O 12	LP*(2)Ga 9	31.11	0.76	0.195
BD (1)Zn 5–O 12	LP*(3)Ga 9	10.89	0.54	0.097
LP*(6)Zn 5	LP*(2)Ga 9	20.41	0.1	0.135
LP (1)Ga 9	BD*(1)Ga 7–O 10	12.89	0.23	0.081
LP (1)Ga 9	BD*(1) O 3–Zn 6	15.37	0.13	0.067

**Table 14:** Second-order perturbation theory analysis of donor–acceptor interactions in the Natural Bond Orbital (NBO) framework for the six water molecules on the Zn_3_O_3_^/^Ga_3_O_3_ cluster.

Donor NBO (i)	Acceptor NBO (j)	E(2), kcal/mol	E(j) − E(i), a.u.	F(i, j), a.u.
LP (3) O 1	RY*(1) H 29	2.24	1.62	0.079
BD*(1) O 1–H 29	RY*(1) H 29	5.36	0.5	0.273
BD (1) O 1–H 29	LP*(9)Zn 4	2.47	0.98	0.063
BD*(1) O 1–H 29	LP*(2)Ga 7	13.29	0.02	0.055
BD*(1) O 2–H 28	RY*(1) H 28	5.32	0.49	0.274
BD (1) O 2–H 28	LP*(9)Zn 4	2.38	0.98	0.062
BD (1) O 2–H 28	LP*(9)Zn 5	3.05	0.97	0.07
BD (1) O 2–H 28	LP*(1)Ga 8	2.64	0.88	0.063
BD*(1) O 3–H 30	RY*(1) H 30	5.31	0.49	0.275
BD (1) O 3–H 30	LP*(9)Zn 5	2.17	0.97	0.059
BD (1) O 3–H 30	LP*(9)Zn 6	3.29	0.96	0.072
LP*(6)Zn 5	RY*(2) O 15	2.41	0.91	0.161
LP*(6)Zn 6	RY*(2) O 13	2.36	0.91	0.155
LP*(7)Zn 6	BD*(1) H 14–O 24	2.17	0.11	0.062
BD (1) O 13–H 19	LP*(7)Zn 6	3.01	0.99	0.071
CR (1) O 13	LP*(7)Zn 6	2.84	19.23	0.303
LP (1) O 13	LP*(8)Zn 6	3.62	0.38	0.048
LP (2) O 13	LP*(7)Zn 6	4.18	0.76	0.072
LP (3) O 13	LP*(6)Zn 6	21.95	0.71	0.164
LP (3) O 13	LP*(7)Zn 6	16.12	0.82	0.147
LP (3) O 13	RY*(2)Zn 6	2.99	1.66	0.092
LP (3) O 13	RY*(4)Zn 6	4.38	5.2	0.198
LP (2) O 13	BD*(1) H 14–O 24	28.97	0.88	0.202
LP (2) O 24	BD*(1) O 3–H 30	26.77	0.89	0.194
BD*(1) H 14–O 24	RY*(1) H 14	5.04	0.47	0.263
LP (1) O 15	LP*(7)Zn 5	2.58	0.46	0.044
LP (2) O 15	LP*(7)Zn 5	2.35	0.67	0.051
LP (2) O 15	LP*(8)Zn 5	2.2	0.7	0.05
LP (3) O 15	LP*(6)Zn 5	24.92	0.72	0.174
LP (3) O 15	LP*(7)Zn 5	8.35	0.7	0.098
LP (3) O 15	LP*(8)Zn 5	6.09	0.73	0.085
LP (3) O 15	RY*(2)Zn 5	2.04	1.29	0.067
LP (3) O 15	RY*(4)Zn 5	4.79	5.48	0.212
LP (2) O 15	BD*(1) H 20–O 26	29.1	0.89	0.204
BD (1) O 17–H 21	LP*(7)Zn 4	2.72	0.97	0.066
LP (1) O 17	LP*(8)Zn 4	4.13	0.39	0.051
LP (2) O 17	LP*(7)Zn 4	2.9	0.75	0.059
LP (2) O 17	LP*(9)Zn 4	3.02	0.69	0.058
LP (3) O 17	LP*(6)Zn 4	23.19	0.7	0.166
LP (3) O 17	LP*(7)Zn 4	13.09	0.78	0.129
LP (3) O 17	RY*(2)Zn 4	2.88	1.54	0.087
LP (3) O 17	RY*(4)Zn 4	4.55	5.4	0.205
LP (2) O 17	BD*(1) H 18–O 22	29.39	0.89	0.205
LP (2) O 22	BD*(1) O 1–H 29	27.38	0.89	0.197
BD*(1) H 18–O 22	RY*(1) H 18	5.05	0.47	0.262
LP (2) O 26	BD*(1) O 2–H 28	26.94	0.89	0.195
BD*(1) H 20–O 26	RY*(1) H 20	5.02	0.47	0.262
BD*(1) H 20–O 26	BD*(1) O 26–H 27	2.03	0.08	0.066

**Table 15:** Second-order perturbation theory analysis of donor–acceptor interactions in the Natural Bond Orbital (NBO) framework for the six water molecules on the isolated 6H_2_O cluster.

Donor NBO (i)	Acceptor NBO (j)	E(2), kcal/mol	E(j) − E(i), a.u.	F(i, j), a.u.
BD*(1) O 1–H 2	RY*(1) H 2	12.44	0.4	0.261
LP (2) O 1	BD*(1) O 10–H 11	66.08	0.94	0.222
BD*(1) O 3–H 8	RY*(1) H 8	12.43	0.4	0.261
LP (2) O 3	BD*(1) O 12–H 13	66.02	0.94	0.222
BD*(1) O 5–H 6	RY*(1) H 6	12.43	0.4	0.261
LP (2) O 5	BD*(1) O 14–H 15	65.91	0.94	0.222
LP (2) O 10	BD*(1) O 5–H 6	66.02	0.94	0.222
LP (2) O 12	BD*(1) O 1–H 2	66.02	0.94	0.222
BD*(1) O 12–H 13	RY*(1) H 13	12.43	0.4	0.261
LP (2) O 14	BD*(1) O 3–H 8	65.92	0.94	0.222
BD*(1) O 14–H 15	RY*(1) H 15	12.43	0.4	0.261

### Electron localization and electrostatic potential analyses

**Fig. 8:**
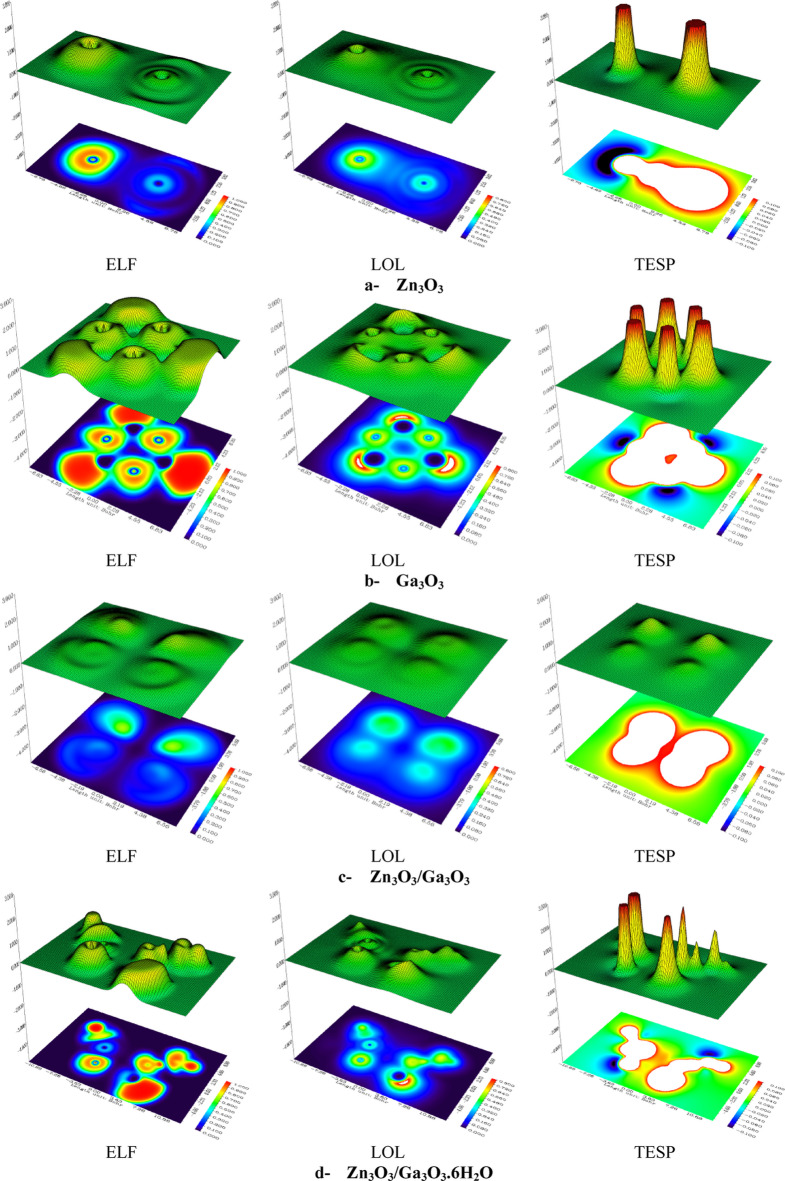
Topological analysis of Electron localization function (ELF) localized orbital locator (LOL) values for Zn_3_O_3_, Ga_3_O_3_,Zn_3_O_3_/Ga_3_O_3_, and Zn_3_O_3_/Ga_3_O_3_.6H_2_O: Electron Localization Function (ELF), Localized Orbital Locator (LOL), and Total Electrostatic Potential (TESP) surfaces.

The ELF and LOL analyses (Figure 8; Tables 16, 17, 18 and 19) reveal a consistent electronic signature across all systems, characterized by complete electron localization at oxygen centers (ELF ≈ LOL ≈ 1.00) and negligible localization at Zn and Ga atoms (ELF ≈ LOL ≈ 0.00), confirming the predominantly ionic nature of the metal–oxygen interactions. In pristine Zn₃O₃ and Ga₃O₃, this localization generates well-defined Lewis basic oxygen sites, while the corresponding TESP surfaces display distinct negative potential regions on oxygen atoms and electropositive metal centers, enabling electrostatic interaction with polar water molecules. Upon formation of the Zn₃O₃/Ga₃O₃ heterostructure, the interfacial coupling induces a more heterogeneous and spatially extended negative electrostatic landscape, effectively increasing the density and accessibility of hydrophilic adsorption sites. Notably, hydration of the composite (Zn₃O₃/Ga₃O₃·6 H₂O) introduces highly localized electronic regions on both oxygen and hydrogen atoms, with slightly reduced LOL values for hydrogen (0.94–0.97), indicative of strong hydrogen-bond participation. The resulting TESP surface exhibits a continuous network of complementary positive and negative regions, promoting cooperative hydrogen bonding and stabilizing adsorbed water molecules. These features collectively demonstrate that interfacial oxide coupling and controlled hydration synergistically enhance surface polarity and hydrogen-bonding capability, providing a rational electronic-structure basis for designing Zn–Ga oxide composites with superior water adsorption and retention performance.

**Table 16:** Electron localization function (ELF) localized orbital locator (LOL) values for Zn_3_O_3_.

CP	Corresponding nucleus	ELF	LOL
1	3(O)	1.00	1.00
2	5(Zn)	0.00	0.00
3	6(Zn)	0.00	0.00
4	2(O)	1.00	1.00
5	1(O)	1.00	1.00
6	4(Zn)	0.00	0.00

**Table 17:** Electron localization function (ELF) localized orbital locator (LOL) values for Ga_3_O_3_.

CP	Corresponding nucleus	ELF	LOL
1	6(O)	1.00	1.00
2	2(Ga)	0.00	0.00
3	3(Ga)	0.00	0.00
4	5(O)	1.00	1.00
5	4(O)	1.00	1.00
6	1(Ga)	0.00	0.00

**Table 18:** Electron localization function (ELF) localized orbital locator (LOL) values for Zn_3_O_3_/Ga_3_O_3_.

CP	Corresponding nucleus	ELF	LOL
1	12(O)	1.00	1.00
2	9(Ga)	0.00	0.00
3	5(Zn)	0.00	0.00
4	3(O)	1.00	1.00
5	8(Ga)	0.00	0.00
6	10(O)	1.00	1.00
7	2(O)	1.00	1.00
8	6(Zn)	0.00	0.00
9	11(O)	1.00	1.00
10	7(Ga)	0.00	0.00
11	4(Zn)	0.00	0.00
12	1(O)	1.00	1.00

**Table 19:** Electron localization function (ELF) localized orbital locator (LOL) values for Zn_3_O_3_/Ga_3_O_3_.6H_2_O.

CP	Corresponding nucleus	ELF	LOL
1	16(H)	1.00	0.97
2	15(O)	1.00	1.00
3	20(H)	1.00	0.94
4	25(H)	1.00	0.97
5	27(H)	1.00	0.97
6	26(O)	1.00	1.00
7	24(O)	1.00	1.00
8	5(Zn)	0.00	0.00
9	30(H)	1.00	0.94
10	3(O)	1.00	1.00
11	14(H)	1.00	0.94
12	28(H)	1.00	0.94
13	12(O)	1.00	1.00
14	9(Ga)	0.00	0.00
15	2(O)	1.00	1.00
16	8(Ga)	0.00	0.00
17	13(O)	1.00	1.00
18	6(Zn)	0.00	0.00
19	19(H)	1.00	0.97
20	10(O)	1.00	1.00
21	4(Zn)	0.00	0.00
22	11(O)	1.00	1.00
23	1(O)	1.00	1.00
24	21(H)	1.00	0.97
25	17(O)	1.00	1.00
26	7(Ga)	0.00	0.00
27	29(H)	1.00	0.94
28	18(H)	1.00	0.94
29	22(O)	1.00	1.00
30	23(H)	1.00	0.97

### Vibrational spectroscopic analysis of Zn₃O₃–Ga₃O₃ composites and hydration effects

**Fig. 9:**
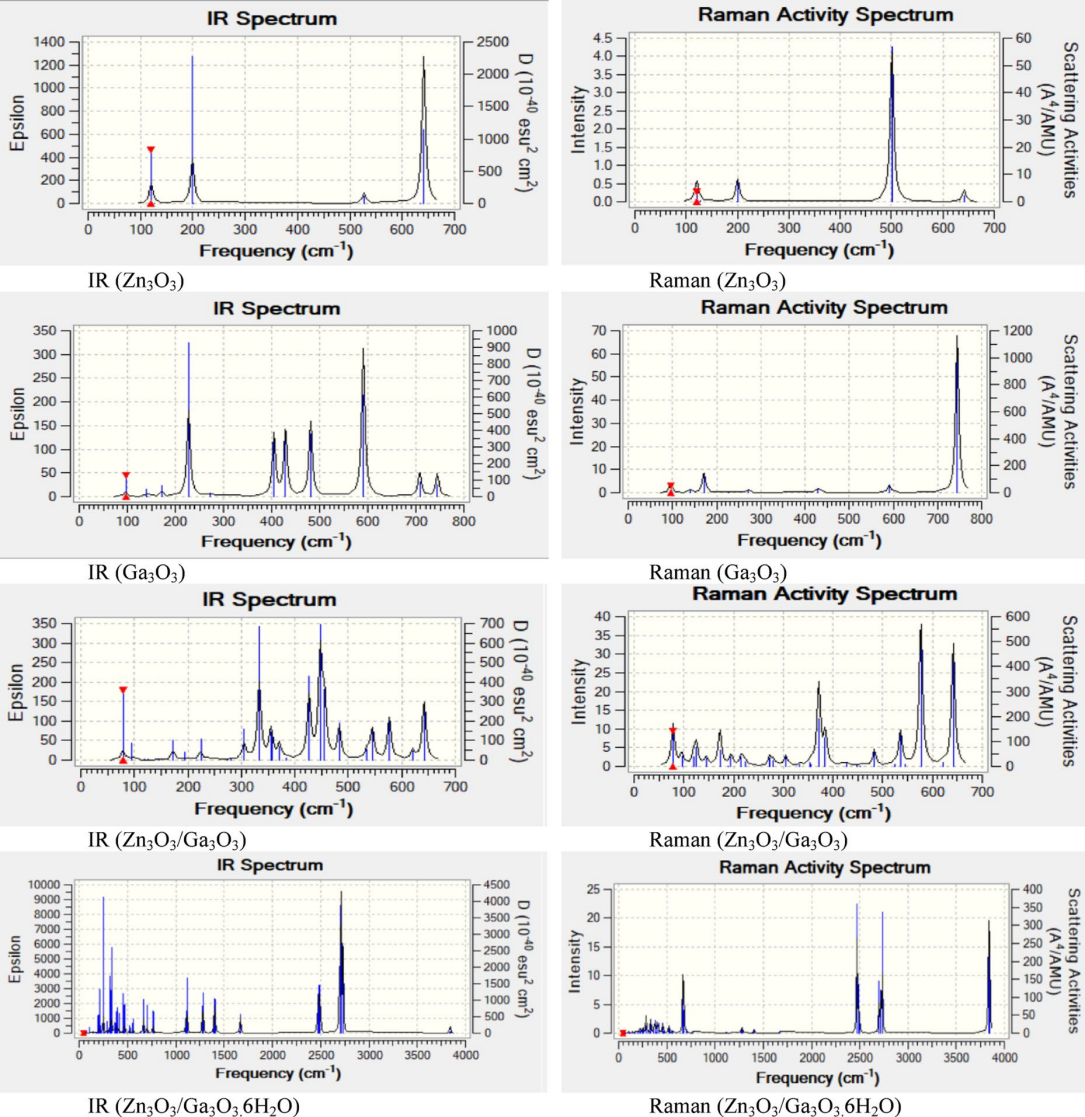
The calculated (IR & Raman) for (**a**) Zn_3_O_3_, (**b**) Ga_3_O_3_ (**c**) Zn_3_O_3_/Ga_3_O_3_ composite and (**d**) Zn_3_O_3_/Ga_3_O_3_ composite interacting with 6 water molecules.

Figure 9 presents the calculated IR & Raman for a- Zn_3_O_3_, b- Ga_3_O_3_ c- Zn_3_O_3_/Ga_3_O_3_ composite and d- Zn_3_O_3_/Ga_3_O_3_ composite interacting with 6 water molecules. As indicated in Figure 8a, the IR of Zn_3_O_3_ shows primary absorption peaks between 100 cm^− 1^ and 250 cm^− 1^, with the most intense peak appearing at approximately 650 cm^− 1^. While the Raman of Zn_3_O_3_ is characterized by a dominant sharp peak at 500 cm^− 1^ and secondary peaks near 150 cm^− 1^ and 200 cm^− 1^. Figure 8b, displays IR bands for Ga_3_O_3_ in the 400–600 cm^− 1^ range, with a significant sharp band below 600 cm^− 1^. While Raman exhibits the most intense scattering activity at approximately 750 cm^− 1^, with a smaller peak near 180 cm^− 1^. The IR of Zn_3_O_3_/Ga_3_O_3_ composite indicated in Figure 8c, shows a combination of features from both individual clusters. Prominent peaks are seen at ~ 100 cm^− 1^, ~ 330 cm^− 1^, and a strong doublet near 450 cm^− 1^. Raman shows features multiple active modes across the 100–700 cm^− 1^ range, with distinct peaks at approximately 380 cm^− 1^, 580 cm^− 1^, and 650 cm^− 1^. As Zn_3_O_3_/Ga_3_O_3_ composite interacting with 6 water molecules the IR shown in Figure 8d a high-frequency region not seen in the dry clusters. Significant peaks appear between 3000 cm^− 1^ and 4000 cm^− 1^ (O–H stretching) and near 1600 cm^− 1^ (H-O-H bending). Raman spectrum shows new activity in the 3000–4000 cm^− 1^ range, correlating with the water interactions, alongside the original framework modes below 1000 cm^− 1^.

The correlation between the IR and Raman data reveals the structural evolution of the composite. The transition from individual clusters to the composite shows a significant redistribution of vibrational modes. The emergence of new bands in the composite’s IR spectrum, which were absent in the isolated clusters, confirms the formation of new chemical bonds and electronic coupling between the Zinc-oxide and Gallium-oxide phases. The systems exhibit a “complementary” relationship where high-intensity IR peaks often correspond to low-intensity Raman peaks and vice versa. This indicates that the clusters maintain a degree of symmetry where certain vibrations cause a large change in dipole moment (IR active) while others primarily change the polarizability (Raman active).

The most striking correlation is found in the hydrated composite. Both the IR and Raman spectra show a new, high-energy vibrational region. These peaks are the definitive signature of O-H stretching modes from the 6 water molecules, confirming that the water has successfully interacted with the composite framework.

Despite the intense new signals from water in the high-frequency range, the “fingerprint” region in the hydrated sample remains largely consistent with the dry composite. This suggests that the structural integrity of the Zn_3_O_3_/Ga_3_O_3_ framework is robust and is not degraded by the presence of moisture.

### Density of states DOS

**Fig. 10:**
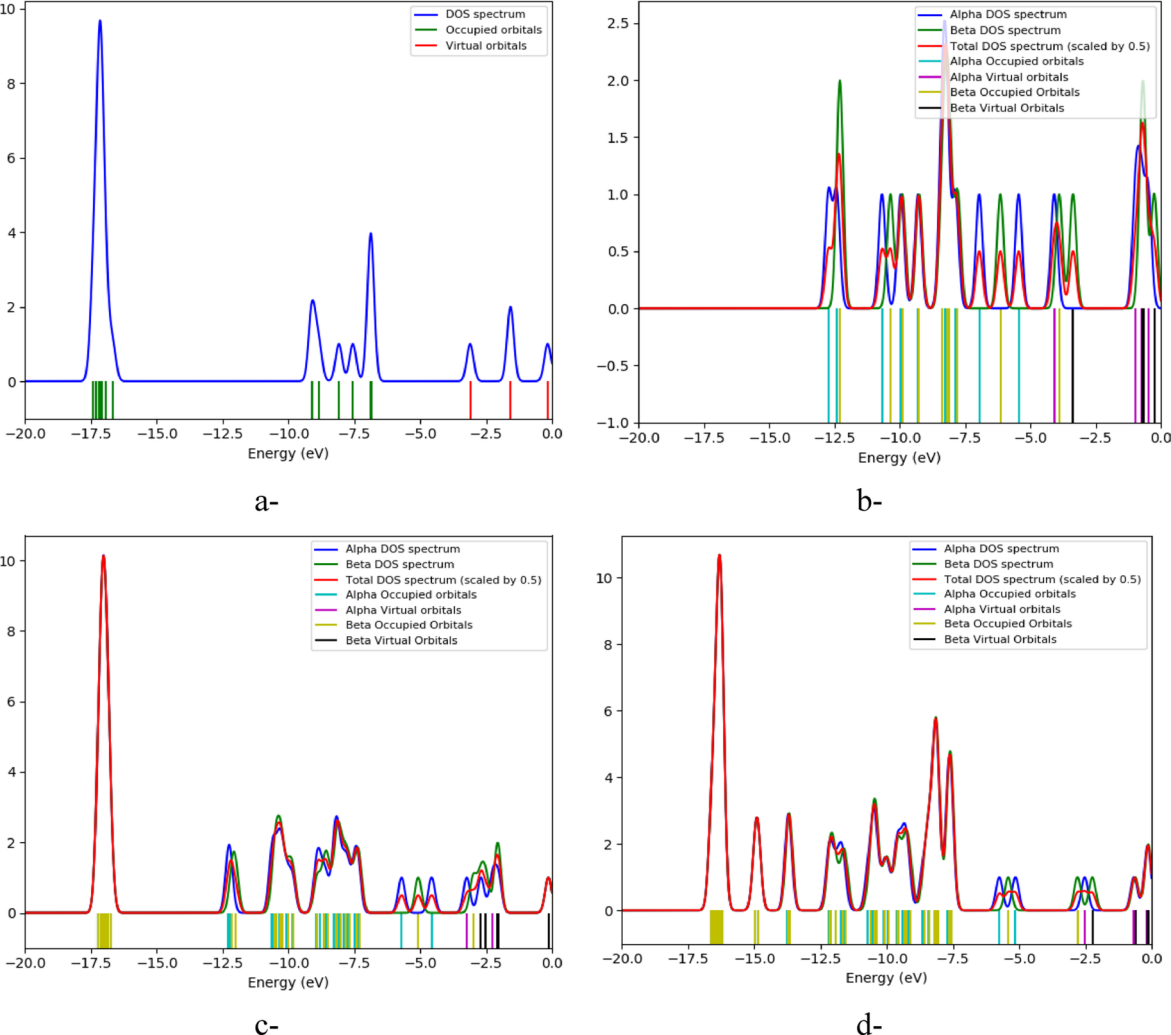
Calculated Density of States DOS for (**a**) Zn_3_O_3_, (**b**) Ga_3_O_3_ (**c**) Zn_3_O_3_/Ga_3_O_3_ composite and (**d**) Zn_3_O_3_/Ga_3_O_3_ composite interacting with 6 water molecules.

Density of States DOS was calculated from Gaussia 09 output files using GaussSum software^51^. Figure 10 presents the calculated Density of States (DOS) for four distinct systems to demonstrate the electronic evolution from individual components to a hydrated composite.

Figure 10a and b establish the baseline electronic states for the isolated clusters. They show the distribution of energy states that define the intrinsic electronic properties such as the band gap of the Zn₃O₃ and Ga₃O₃ building blocks.

Figure 9c shows that, the clusters merge into the composite, the DOS plots show a modification in the energy state distribution. This confirms the successful interfacial electronic coupling between the Zn₃O₃ and Ga₃O₃ domains, which results in a narrowed band gap (1.3312 eV).

Finally, Figure 9d demonstrates the impact of environmental moisture. The DOS for this system shows a shift in the energy states, correlating with the observed increase in the HOMO–LUMO energy gap (to 2.3392 eV) upon water adsorption.

The DOS results in Table 4 emphasize that the DOS plots provide the fundamental electronic evidence for the system’s sensitivity to its environment.

By explicitly linking the shifting and broadening of the energy peaks observed in the DOS plots (Figure 10a, b, c & d) to the calculated changes in the HOMO–LUMO energy gap which increases from 1.33 eV in the pristine composite to 2.34 eV upon the adsorption of six water molecules it is demonstrated that the electronic properties of the system are highly tunable. This correlation confirms that the hydration of the Zn₃O₃/Ga₃O₃ interface significantly alters the charge carrier distribution, thereby justifying the composite’s potential as a responsive active surface material. Providing this direct narrative bridge, where the visual data of the DOS plots validates the quantitative shifts in the electronic descriptors, offers a robust explanation of how interfacial interactions with water molecules modulate the composite’s overall electronic structure.

### Scope and limitations of the present study

While this study provides crucial atomistic views into the electronic coupling and early water-absorption mechanisms of the Zn_3_O_3_/Ga_3_O_3_ composite, some structural and computational boundaries should really be kept in mind, kind of, even though the main story looks clean.

1. Model Dimensionality and Size Effects: Here we rely on localized molecular cluster models (Zn_3_O_3_, Ga_3_O_3_, plus their mixed composite) simulated using localized basis sets (LANL2DZ) inside a vacuum setting. Cluster approaches are excellent for chasing in-the-moment chemical happenings, electrostatic potential landscapes (MESP), and weak non-covalent contacts (NCI) during the first water capture. Still, these clusters do not fully mimic the macroscopic bulk behaviour, any surface rearrangements, or the periodic edge motifs that come with extended 2D or 3D heterostructures. So, it’s not really a full representation of the extended system, it’s more like a focused snapshot.
2. Thermodynamic Band-Edge Alignment: The electronic outcomes in this work are centered on molecular orbital limits, meaning the HOMO–LUMO separation, and on local density of states (DOS) to discuss interfacial charge transfer. A rigorous and fully absolute band-edge positioning relative to the standard hydrogen electrode (NHE), or equivalently the vacuum level, would need an explicit solvent description and macroscopic space-charge layers. That sort of treatment is beyond the localized cluster capacity of this paper. Therefore, the “alignment” situations shown here are mainly descriptive of molecular-level orbital intermixing, not macro-scale thermodynamic boundaries that control bulk water-splitting cell performance.
3. Reaction Kinetics and Overpotentials: This research traces stable adsorption arrangements and how the electronic structure responds when water is present on the composite. But a full kinetic route map for the Hydrogen Evolution Reaction (HER) and the Oxygen Evolution Reaction (OER) requires following several very fragile transition structures, computing explicit activation energy barriers, and locating reactive intermediates that can be short-lived. Those kinetic tracks, and the associated overpotentials, are very sensitive to changing surface coverage as well as solvent effects. Capturing that reliably takes a broad and demanding periodic transition-state sampling, which is not included in the current cluster framework, at least not in a comprehensive way.

Consequently, the results laid out here ought to be taken as a bit of a starting blueprint, kind of mapping the basic interfacial chemistry, charge polarization, and the early-stage water affinity tied to the Zn_3_O_3_/Ga_3_O_3_ heterostructure. But fully pulling apart the macro-scale thermodynamic band alignments, alongside the explicit HER/OER kinetic reaction coordinates, is still an extremely necessary next move, and it will be handled in later studies. Those later efforts will use periodic boundary conditions PBC and explicit solvent modeling.

## Conclusions

The first-principal investigation confirms that the Zn_3_O_3_/Ga_3_O_3_ composite is a highly reactive and tuneable nanostructure suitable for water-splitting applications. Key conclusions from the study include.

**Electronic Sensitivity**: The composite exhibits a narrow energy gap (1.3312 eV), which increases significantly upon interaction with water (2.3392 eV), suggesting that the material must be protected from moisture to maintain specific high-performance electronic characteristics.

**Bonding and Stability**: QTAIM and NCI analyses indicate that the framework’s stability is governed by highly polarized ionic/coordination bonds and a balance of steric repulsion and weak dispersive interactions.

**Water Interaction**: The composite shows a strong affinity for water, with adsorption driven by electrostatic complementarity between electron-rich oxygen sites and electron-deficient metal (Zn and Ga) centers.

**Structural Integrity**: Despite the formation of new O–H stretching modes in hydrated samples, the “fingerprint” vibrational region remains consistent, indicating that the Zn_3_O_3_/Ga_3_O_3_ framework is robust and maintains its structural integrity in the presence of water. These vibrational modes (the O–H stretching) could explicitly link to the catalytic activation process.

While this study utilizes a cluster-based model Zn_3_O_3_/Ga_3_O_3_ to analyze the interfacial electronic properties and water adsorption mechanisms, it is important to acknowledge that this approach primarily targets the local electronic environment rather than the long-range periodicity of extended crystalline surfaces. Nevertheless, the local coordination of the Zn-Ga active sites and the nature of the chemical bonding (as revealed by NBO, QTAIM, and NCI analyses) provide fundamental insights into the catalytic activation of water at the nanoscale. These findings serve as a robust molecular benchmark, capturing the essential donor-acceptor interactions that dictate interfacial charge redistribution. Future research employing periodic slab calculations could build upon these results to evaluate how long-range lattice effects influence collective carrier transport across the interface.

The calculated DOS confirms that the Zn₃O₃/Ga₃O₃ composite possesses a highly tunable electronic structure, establishing it as a promising candidate for high-efficiency photocatalytic water-splitting applications.

The interaction of the Zn₃O₃/Ga₃O₃ composite with a 6-water cluster induces a significant increase in the HOMO-LUMO energy gap, suggesting that moisture can be used to tune the material’s electronic reactivity. This process is stabilized by a synergistic network of metal-oxygen coordination and hydrogen bonding, which allows for water adsorption and dissociation without degrading the structural integrity of the composite framework.

## Data Availability

The data that support the findings of this study are available from the corresponding author upon reasonable request.

## References

[1] Xu, R. et al. Non-noble nickel-modified covalent organic framework for photocatalytic hydrogen production. *Colloids Surf., A*. **706**, 135792. 10.1016/j.colsurfa.2024.135792 (2025).

[2] Yang, Q. et al. Rational construction of a ternary ZnO/In₂S₃/ZnIn₂S₄ dual S-scheme heterojunction for efficient photocatalytic hydrogen evolution. *Appl. Surf. Sci.* **726**, 165910. 10.1016/j.apsusc.2026.165910 (2026).

[3] Mao, D. et al. Monolayer YTeI for photocatalytic water splitting. *Appl. Surf. Sci.* **710**, 163928. 10.1016/j.apsusc.2025.163928 (2025).

[4] Zhang, C. et al. Janus MSiGeZ₄ as an efficient photocatalyst for visible-light driven spontaneous oxygen evolution reaction: High-throughput screening and DFT calculations. *Chem. Eng. J.* **525**, 170686. 10.1016/j.cej.2025.170686 (2025).

[5] Mallick, P., Sahoo, S. K. & Satpathy, S. K. Different strategies to improve photocatalytic activity of graphitic carbon nitride (g-C₃N₄) semiconductor nanomaterials for hydrogen generation. *J. Mol. Liq.* **406**, 125071. 10.1016/j.molliq.2024.125071 (2024).

[6] Rocha, L. S. R. et al. Photoluminescence emission at room temperature in zinc oxide nano-columns. *Mater. Res. Bull.* **50**, 12–17. 10.1016/j.materresbull.2013.09.049 (2014).

[7] Zeng, C. et al. Modulation of dielectric and antibacterial properties of Zn₀.₅Mn₀.₅O nanoparticles by post growth annealing method. *Heliyon* **10** (16), e36035. 10.1016/j.heliyon.2024.e36035 (2024). 39247313 10.1016/j.heliyon.2024.e36035 PMC11379577

[8] Shokri, A. & Amirmazlaghani, M. Experimental and simulation study of ZnO nanowire/Si heterojunctions as radioisotope batteries for long-term micro-power applications. *Results Phys.* **80**, 108569. 10.1016/j.rinp.2025.108569 (2026).

[9] Mändl, S. & Rauschenbach, B. Formation of transparent ZnO layers by MePIIID. Nuclear instruments and methods. *Phys. Res. Sect. B: Beam Interact. Mater. Atoms*. **242** (1–2), 293–295. (2006). 10.1016/j.nimb.2005.08.038 (2006).

[10] Litimein, F., Rached, D., Khenata, R. & Baltache, H. FPLAPW study of the structural, electronic, and optical properties of Ga₂O₃: Monoclinic and hexagonal phases. *J. Alloys Compd.* **488** (1), 148–156. 10.1016/j.jallcom.2009.08.092 (2009).

[11] Zhang, R. et al. Modulating electronic properties of β-Ga₂O₃ by strain engineering. *Results Phys.* **52**, 106916. 10.1016/j.rinp.2023.106916 (2023).

[12] Liu, T. et al. Orientation-dependent surface radiation damage in β-Ga₂O₃ explored by atomistic simulations. *Acta Mater.* **300**, 121484. 10.1016/j.actamat.2025.121484 (2025).

[13] Rajabathar, J. R., Munusamy, M. A. & Al-Lohedan, H. A. Preparation, textural and photoluminescence characterization of green fluorescence protein-immobilised Ga-ZnO (GZO)-nanocomposites. *J. Photochem. Photobiol., B*. **165**, 202–212. 10.1016/j.jphotobiol.2016.10.028 (2016). 27816642 10.1016/j.jphotobiol.2016.10.028

[14] Safeera, T. A. & Anila, E. I. An investigation on the luminescence quenching mechanism of ZnGa₂O₄:Tb³⁺ phosphor. *J. Lumin.* **205**, 277–281. 10.1016/j.jlumin.2018.09.033 (2019).

[15] Bashir, Z., Kayani, Z. N., Waseem, S., Riaz, S. & Naseem, S. Synthesis and characterization of InGaZnO nanocomposites: an insight of optical, dielectric, and magnetic properties. *Optik* **319**, 172094. (2024). 10.1016/j.ijleo.2024.172094 (2024).

[16] Tuo, Y. et al. Advances in Cu-based catalysts for methanol steam reforming: mechanistic insights and atomic-level design. *J. Energy Chem.* **112**, 64–89. (2026). 10.1016/j.jechem.2025.08.032 (2026).

[17] Zhang, R., Villanueva, A., Alamdari, H. & Kaliaguine, S. Crystal structure, redox properties and catalytic performance of Ga-based mixed oxides for NO reduction by C₃H₆. *Catal Commun.* **9** (1), 111–116. 10.1016/j.catcom.2007.05.029 (2008).

[18] Lawes, N. et al. CO₂ hydrogenation to methanol on intermetallic PdGa and PdIn catalysts and the effect of Zn co-deposition. *Appl. Catal. A*. **679**, 119735. 10.1016/j.apcata.2024.119735 (2024).

[19] Subki, A. S. R. A. et al. Effects of varying the amount of reduced graphene oxide loading on the humidity sensing performance of zinc oxide/reduced graphene oxide nanocomposites on cellulose filter paper. *J. Alloys Compd.* **926**, 166728. 10.1016/j.jallcom.2022.166728 (2022).

[20] Talwar, M. N., Shirni, A. P., Kumar, R. T. R. & Prakash, A. P. G. Highly selective ammonia gas sensor using Ga₂O₃/MoO₃ nanocomposite at ambient atmospheric conditions. *Mater. Sci. Eng. B 324(Part B)*. (2026). 10.1016/j.mseb.2025.119027 (2026).

[21] Huang, C. Y., Wei, C. T., Hsin, C. L. & Huang, C. W. Fabrication and characterization of two-dimensional Zn-doped Ga₂O₃-based oxide films using liquid metal alloys. *Surf. Interfaces*. **75**, 107753. 10.1016/j.surfin.2025.107753 (2025).

[22] Wen, H. et al. Effect of Ga doping on the catalytic performance of Zn-ZSM-5 for CO₂-assisted oxidative dehydrogenation of C₂H₆. *J. CO₂ Utilization*. **95**, 103066. 10.1016/j.jcou.2025.103066 (2025).

[23] Xing, M. et al. Role of Ga₂O₃ crystallite in OX-ZEO catalyst for catalytic CO₂ hydrogenation to light olefins. *Chem. Eng. J.* **520**, 166177. 10.1016/j.cej.2025.166177 (2025).

[24] Elhaes, H., Ibrahim, A., Osman, O. & Ibrahim, M. A. Molecular modeling analysis for functionalized graphene/sodium alginate composite. *Sci. Rep.* **14**, 14825. 10.1038/s41598-024-64698-x (2024). 38937511 10.1038/s41598-024-64698-x PMC11211416

[25] Elhaes, H. & Ibrahim, M. A. Investigating the electronic properties of graphene oxide functionalized with benzoic acid. *Sci. Rep.* **15**, 38105. 10.1038/s41598-025-22839-w (2025). 41174006 10.1038/s41598-025-22839-w PMC12578904

[26] Ibrahim, A., Elhaes, H., Khaled, N. A. & Ibrahim, M. A. On the analyses of graphene oxide/polypyrrole/zinc oxide nanocomposites. *Sci. Rep.* **15**, 34284. 10.1038/s41598-025-20194-4 (2025). 41034491 10.1038/s41598-025-20194-4 PMC12488988

[27] El-Sayed, N. M., Elhaes, H., Ibrahim, A. & Ibrahim, M. A. Investigating the electronic properties of edge glycine/biopolymer/graphene quantum dots. *Sci. Rep.* **14**, 21973. 10.1038/s41598-024-71655-1 (2024). 39304667 10.1038/s41598-024-71655-1 PMC11415379

[28] Pandey, A., Scherich, H. & Drabold, D. A. Density functional theory model of amorphous zinc oxide (a-ZnO) and a-X₀.₃₇₅Z₀.₆₂₅O (X = Al, Ga and In). *J. Non-cryst. Solids*. **455**, 98–101. 10.1016/j.jnoncrysol.2016.10.035 (2017).

[29] Meshina, K. et al. Zinc oxide nanoobjects for dye removal: effective photocatalyst design via oriented attachment process. *Surf. Interfaces*. **60**, 106006. (2025). 10.1016/j.surfin.2025.106006 (2025).

[30] Eshonqulov, G. B., Meyliyeva, A. A., Yuldashev, S. U. & Berdiyorov, G. R. Enhanced optical properties of β-Ga₂O₃₋ₓSₓ: A DFT study. *Phys. B: Condens. Matter*. **714**, 417367. 10.1016/j.physb.2025.417367 (2025).

[31] Harun, K., Salleh, N. A., Deghfel, B., Yaakob, M. K. & Mohamad, A. A. DFT + U calculations for electronic, structural, and optical properties of ZnO wurtzite structure: A review. *Results Phys.* **16**, 102829. 10.1016/j.rinp.2019.102829 (2020).

[32] Wan, J. et al. Band alignment of ZnO-based nanorod arrays for enhanced visible light photocatalytic performance. *RSC Adv.* **12** (42), 27189–27198. 10.1039/D2RA03940K (2022). 36276038 10.1039/d2ra03940k PMC9511231

[33] Tezekbay, Y. et al. Photocatalytic optimization of ZnO–Ga₂O₃ composite thin films for PEC water splitting: effects of thickness, environment, and annealing temperature. *RSC Adv.* **15** (34), 27586–27593. (2025). 10.1039/D5RA03463A (2025). 40761900 10.1039/d5ra03463a PMC12320224

[34] Abdullah, R. et al. Recent advances in zinc oxide-based photoanodes for photoelectrochemical water splitting. *Int. J. Hydrog. Energy*. **107**, 183–207. 10.1016/j.ijhydene.2024.05.461 (2025).

[35] Gueddouch, K. et al. Structural, optical and electrical properties of F doped ZnO films: experimental and theoretical study. *Mater. Today: Proc.* **66(Part 1)**, 151–157. (2022). 10.1016/j.matpr.2022.04.293 (2022).

[36] Khan, I. et al. Single-step strategy for the fabrication of GaON/ZnO nanoarchitectured photoanode their experimental and computational photoelectrochemical water splitting. *Nano Energy*. **44**, 23–33. 10.1016/j.nanoen.2017.11.050 (2018).

[37] Frisch, M. J. et al. *Gaussian 09, Revision C.01* (Gaussian, Inc, 2010).

[38] Dennington, R., Keith, T. A., Millam, J. M. & GaussView *Version 6* (Semichem Inc., 2016).

[39] Becke, A. D. Density-functional thermochemistry. I. The effect of the exchange-only gradient correction. *J. Chem. Phys.* **96** (3), 2155–2160. 10.1063/1.462066 (1992).

[40] Petersson, G. A. & Al-Laham, M. A. A complete basis set model chemistry. II. Open-shell systems and the total energies of the first-row atoms. *J. Chem. Phys.* **94** (9), 6081–6090. 10.1063/1.460447 (1991).

[41] Lee, C., Yang, W. & Parr, R. G. Development of the Colle–Salvetti correlation-energy formula into a functional of the electron density. *Phys. Rev. B*. **37** (2), 785–789. 10.1103/PhysRevB.37.785 (1988). 10.1103/physrevb.37.785 9944570

[42] Glendening, F. W. E., Badenhoop, J., Reed, A. & Carpenter, J. *NBO 3.1* (Theoretical Chemistry Institute, University of Wisconsin, 1996).

[43] Ortiz, P. D. et al. A novel series of pyrazole derivatives toward biological applications: Experimental and conceptual DFT characterization. *Mol. Diversity*. **26**, 2443–2457. 10.1007/s11030-021-10342-z (2022). 10.1007/s11030-021-10342-z 34724138

[44] El-Taib Heakal, F., Attia, S. K., Rizk, S. A., Abou Essa, M. A. & Elkholy, A. E. Synthesis, characterization and computational chemical study of novel pyrazole derivatives as anticorrosion and antiscalant agents. *J. Mol. Struct.* **1147**, 714–724. 10.1016/j.molstruc.2017.07.006 (2017).

[45] Cortés-Guzmán, F. & Bader, R. F. W. Complementarity of QTAIM and MO theory in the study of bonding in donor–acceptor complexes. *Coord. Chem. Rev.* **249** (5), 633–662. 10.1016/j.ccr.2004.08.022 (2005).

[46] Johnson, E. R. et al. Revealing noncovalent interactions. *J. Am. Chem. Soc.* **132** (18), 6498–6506. 10.1021/ja100936w (2010). 20394428 10.1021/ja100936w PMC2864795

[47] Lu, T. (ed) (n.d.). Plotting electrostatic potential colored molecular surface map with ESP surface extrema via Multiwfn and VMD.

[48] Lu, T. & Chen, F. Multiwfn: A multifunctional wavefunction analyzer. *J. Comput. Chem.* **33** (5), 580–592. 10.1002/jcc.22885 (2012). 22162017 10.1002/jcc.22885

[49] Humphrey, W., Dalke, A. & Schulten, K. VMD: visual molecular dynamics. *J. Mol. Graph.* **14**, 33–38. (1996). 10.1016/0263-7855(96)00018-5 (1996). 8744570 10.1016/0263-7855(96)00018-5

[50] Abbasi, A. & Khataee, A. Band gap tunability and structural stability of metal/nonmetal codoped group-IV tin nanotubes: effect of spin-orbit coupling. *Phys. E: Low-Dimens. Syst. Nanostruct.* **114**, 113644. (2019). 10.1016/j.physe.2019.113644 (2019).

[51] O’Boyle, N. M., Tenderholt, A. L. & Langner, K. M. A Library for package-independent computational chemistry algorithms. *J. Comp. Chem. 2008*. **29**, 839–845. (2008). 10.1002/jcc.20823 (2008). 10.1002/jcc.20823 17849392

